# Assisting Forearm Function in Children With Movement Disorders *via* A Soft Wearable Robot With Equilibrium-Point Control

**DOI:** 10.3389/frobt.2022.877041

**Published:** 2022-06-15

**Authors:** Jonathan Realmuto, Terence D. Sanger

**Affiliations:** ^1^ Department of Mechanical Engineering, University of California, Riverside, Riverside, CA, United States; ^2^ Department of Electrical Engineering and Computer Science, University of California, Irvine, Irvine, CA, United States; ^3^ Children’s Hospital of Orange County, Orange, CA, United States

**Keywords:** wearable robotics, soft robotics, human–robot interaction, reflexes, equilibrium-point control, proprioception

## Abstract

Wearable robots are envisioned to amplify the independence of people with movement impairments by providing daily physical assistance. For portable, comfortable, and safe devices, soft pneumatic-based robots are emerging as a potential solution. However, due to the inherent complexities, including compliance and nonlinear mechanical behavior, feedback control for facilitating human–robot interaction remains a challenge. Herein, we present the design, fabrication, and control architecture of a soft wearable robot that assists in supination and pronation of the forearm. The soft wearable robot integrates an antagonistic pair of pneumatic-based helical actuators to provide active pronation and supination torques. Our main contribution is a bio-inspired equilibrium-point control scheme for integrating proprioceptive feedback and exteroceptive input (e.g., the user’s muscle activation signals) directly with the on/off valve behavior of the soft pneumatic actuators. The proposed human–robot controller is directly inspired by the equilibrium-point hypothesis of motor control, which suggests that voluntary movements arise through shifts in the equilibrium state of the antagonistic muscle pair spanning a joint. We hypothesized that the proposed method would reduce the required effort during dynamic manipulation without affecting the error. In order to evaluate our proposed method, we recruited seven pediatric participants with movement disorders to perform two dynamic interaction tasks with a haptic manipulandum. Each task required the participant to track a sinusoidal trajectory while the haptic manipulandum behaved as a Spring-Dominate system or Inertia-Dominate system. Our results reveal that the soft wearable robot, when active, reduced user effort on average by 14%. This work demonstrates the practical implementation of an equilibrium-point volitional controller for wearable robots and provides a foundational path toward versatile, low-cost, and soft wearable robots.

## 1 Introduction

The ability to manipulate and interact with the environment through one’s upper extremities is fundamental to physical health and overall well-being. Cerebral palsy, the most common cause of serious physical disability in childhood, has no known curative treatment (Morris, 2007; Maenner et al., 2016) and can severely limit physical mobility. Secondary conditions are common and extend into adulthood, including pain and musculoskeletal problems (Murphy et al., 1995; Schwartz et al., 1999), often a consequence of abnormal movements and straining to manage daily life (Ando and Ueda, 2000). While emerging wearable robots are envisioned to provide long-term daily physical assistance for people with mobility impairments (Pons, 2008) and could therefore become a primary treatment for cerebral palsy and other movement disorders, they are not yet widely available.

Among the emerging technologies, advances in soft pneumatic-based actuators are enabling a new generation of lightweight, compliant, and versatile wearable robots (Koh et al., 2017; Simpson et al., 2017; Cappello et al., 2018; Sridar et al., 2018; Realmuto and Sanger, 2019; Zhu et al., 2020; Zhou et al., 2021; Zhu et al., 2022). This new generation of wearable soft robots solves some of the fundamental limitations of rigid robotic structures, including non-portability (Maciejasz et al., 2014), kinematic incompatibilities when the robot and human joints are misaligned (Jarrassé and Morel, 2011), and requiring compensatory non-physiological muscle strategies during movement due to added inertia (Laut et al., 2016). However, no single control strategy has emerged for providing volitionally controlled assistance that allows the robot to adapt in synchrony with the human user under changing environmental requirements.

Unique constraints due to the structure and morphology of soft pneumatic actuators, including limited bandwidth and high compliance, prohibit the use of advanced control techniques designed for physical interaction. For traditionally engineered systems that leverage high-bandwidth DC motors, the impedance control framework provides the theoretical machinery for designing stable interactive behavior (Hogan, 1985; Lee and Hogan, 2016; Hogan and Buerger, 2018; Hogan, 2021). The framework has been successfully deployed for a variety of interactive applications, including industrial robots (Lopes and Almeida, 2008), legged robots (Semini et al., 2015), haptic displays (Mehling et al., 2005), rehabilitation robots (Jamwal et al., 2016), and lower limb wearable robots (Rajasekaran et al., 2015). Although an attractive framework for dealing with physical interaction, impedance control requires high sampling rates and rigid links with high-bandwidth motors to properly render desired impedance (Colgate and Brown, 1994). Soft pneumatic actuators achieve motions through the injection of compressed air (Chou and Hannaford, 1996; Daerden and Lefeber, 2002; Niiyama et al., 2015; Nesler et al., 2018; Zhang et al., 2019; Veale et al., 2021) and are therefore not a good candidate for impedance control in the traditional sense. However, because soft pneumatic actuators can change their intrinsic compliance, they have the ability for direct impedance modulation, meaning that the actuator itself can be modulated to the required impedance for physical interaction. While initials solutions exist (Hajian et al., 1997; Ariga et al., 2012b; Paoletti et al., 2017; Slightam et al., 2018), a generalized approach for impedance modulation has not yet emerged.

The inherent compliance of soft pneumatic actuators allows for a safe and comfortable physical interaction between the user and the robot, but also a large degree of complexity in sensing and control. Emerging devices have limited control capabilities, with many operating in binary modes of assistance/no assistance (Simpson et al., 2017; Cappello et al., 2018; Zhou et al., 2021), or leveraging closed-loop bang–bang or model-based control to maintain a desired internal pressure (Skorina et al., 2015; Koh et al., 2017; Sridar et al., 2018; Chen C. et al., 2020; Park et al., 2020) where the desired state is inferred from other external sensors. To the degree possible, in order to minimize space, complexity, and manufacturing cost, it is desirable to minimize the need for sophisticated electromechanical sensors, motivating new techniques for perception. In fact, inherent compliance can be leveraged for proprioception, that is, an intrinsic sense of the kinematic configuration, thereby reducing the need for other sensor modalities (Wirekoh et al., 2019; Chen W.-H. et al., 2020; Park et al., 2021). Since external forces change the volume (therefore the internal pressure) of the actuator, a pressure sensor can readily detect the interaction. However, if the internal pressure is continuously regulated, it becomes difficult to distinguish physical interactions with the environment from the self-induced variations of the controller, requiring a sophisticated estimator to model the complex dynamics of the soft actuator (Georgiou and Lindquist, 2013). Because of the inherent compliance of soft pneumatic actuators, which behave in a similar fashion to muscle, there should be a clear nexus with biological sensorimotor principles.

Our main idea is that the equilibrium-point hypothesis of motor control provides a framework for facilitating physical interaction with soft pneumatic actuators. The hypothesis posits that voluntary movement results from shifts in the equilibrium angle of a joint, and a combination of the stretch reflex with the passive mechanical properties of the muscles provides restoring torques if the joint angle deviates from the equilibrium point (Feldman, 1966; Feldman, 1986; Flash, 1987; Feldman and Levin, 2009; Latash, 2010). In this way, the equilibrium point represents a point attractor that the stretch reflex drives the joint toward, and a sequence of such equilibria produce movement. The key benefits of harnessing equilibrium-point control for robots are the ability to control the equilibrium configuration independently and joint impedance (e.g., variable impedance behavior) and in a simplified (parametric) manner. Control techniques inspired by the equilibrium-point hypothesis have been investigated in robot walking (Chen et al., 2021), pedaling (Watanabe et al., 2020), opening doors and drawers (Jain and Kemp, 2010), human neuromuscular surrogate models (Lenzi et al., 2011), and controlling antagonist soft actuators (Ariga et al., 2012a; Ariga et al., 2012b).

The aim of this work is to investigate equilibrium-point control for use in wearable robots. To leverage the equilibrium-point idea, we harness the passive properties of antagonistically arranged soft actuators to provide restoring torques if the joint angle deviates from the desired equilibrium point rather than continuously regulating the internal pressure. When sufficient environment interaction or movement intention is detected, a reflex is triggered, which shifts the equilibrium point in the direction of interaction or intention, thereby providing assistive torques in that direction. The proposed method is an instantiation of asynchronous and event-driven control (Aström, 2008). Our current focus is on modulating the equilibrium point and not yet full impedance modulation, and therefore, the proposed wearable robot has an approximately constant net stiffness. However, if proven successful, the method provides the necessary framework for achieving stiffness modulation. We test the proposed method in experiments with human participants using a soft wearable robot designed to provide pronation and supination torques across the forearm, which is an essential degree of freedom in many activities of daily living (Morrey et al., 1981). Our central hypothesis is that the equilibrium-point controller will reduce the required effort to complete dynamic tracking tasks while not affecting the tracking error.

## 2 Materials and Methods

### 2.1 Equilibrium-Point Controller

#### 2.1.1 Problem Statement

**FIGURE 1:**
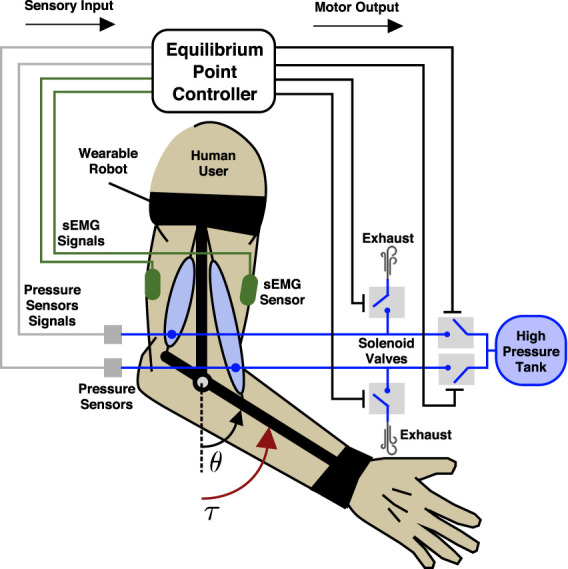
Schematic representation of an elbow assist wearable robot used as an idealized model to conceptually demonstrate our proposed equilibrium-point controller. The wearable robot exerts a torque *τ* about the elbow joint with angle *θ*. The wearable robot consists of two antagonistic soft pneumatic actuators and a support structure and is attached across the human user’s elbow joint. The soft actuators are driven through solenoid valves, one for high pressure and one for exhaust. Pressure sensors monitor the internal pressure of each pneumatic actuator, while two sEMG sensors monitor the agonist (bicep) and antagonist (tricep) biological muscles. The research problem we aim to solve is given the sensory inputs (internal pressure and sEMG) to choose the appropriate motor outputs (valve on/off states) in order to provide physical assistance to the user.

The problem is conceptually illustrated in Figure 1 and consists of choosing the appropriate on/off valve states of the soft pneumatic actuators to provide physical assistance to the user. The two actuators are arranged antagonistically across the human joint, and each one is connected to a pair of solenoid valves: one for high pressure and one for exhaust. The perception of the robot includes the internal pressures of each pneumatic actuator and the surface electromyographic (sEMG) signals corresponding to the biological agonist and antagonist. The problem is mapping the pressure and sEMG signals (sensory inputs) into the solenoid valve states (motor outputs). Note that the illustrative model depicts an elbow-based wearable robot with contractile pneumatic actuators to more clearly communicate the essential idea. However, the forearm robot used in the subsequent study uses helical actuators that operate under the same mechanical principles.

#### 2.1.2 Biological Inspiration

**FIGURE 2:**
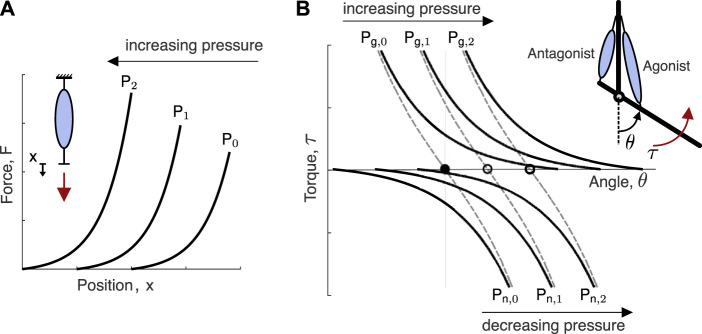
Conceptual overview of the equilibrium-point hypothesis applied to soft pneumatic actuators. **(A)** Idealized force-deflection curves for three different nominal internal pressures of a contraction-type soft actuator. Nominal pressure refers to the internal pressure before the external load is applied, which invariably changes the internal pressure do to volume changes. As the nominal pressure of the actuator is increased, the force-deflection curve is shifted. **(B)** Idealized force-deflection curves for antagonistically configured contraction-type soft pneumatic actuator for three different nominal internal pressures demonstrate a shift in the equilibrium position of the joint, while the net force-deflection curve (dashed traces) is maintained constant. The depicted shift in equilibrium occurs by increasing the agonist actuator’s internal pressure (*P*_*g*,0_ < *P*_*g*,1_< *P*_*g*,2_) and decreasing the antagonist actuator’s internal pressure (*P*_*g*,0_ > *P*_*g*,1_ > *P*_*g*,2_).

The main idea of our approach is to leverage the passive stiffness properties of the soft actuators to produce stable equilibria, which are then shifted to assist the user. To understand the concept, first, consider a single contractile pneumatic actuator (Figure 2A). When pressurized to a nominal pressure *P*_0_, the actuator behaves as a (nonlinear) spring under loading. If the nominal pressure is increased to *P*_1_, the stiffness curve is shifted so that more force is required to produce the same end-point displacement. If two pneumatic actuators are configured antagonistically across a joint and inflated to the same nominal pressure (*P*_*g*,0_ for the agonist, and *P*_*n*,0_ for the antagonist), the opposing torques will produce an equilibrium-point (at *θ* = 0) where the net torque is zero (Figure 2B). If the agonist’s internal pressure is increased to *P*_*g*,1_ > *P*_*g*,0_ and the antagonist is deflated to *P*_*n*,1_ < *P*_*n*,0_, then the equilibrium point will shift in the direction of the agonist. If the stiffness characteristics are invariant to shifts in nominal pressures, the net stiffness will remain identical in both configurations (seen as the dashed traces in Figure 2B). While biological muscles under control of their stretch reflex have invariant characteristic stiffness curves (Gottlieb and Agarwal, 1988), pneumatic actuators do not (Ariga et al., 2012a). Therefore, the conceptual model in Figure 2 is an idealization. In practice, the net stiffness would change according to the intrinsic properties of the actuators. Nevertheless, the shifting of the equilibria still applies.

#### 2.1.3 Triggered Shifts in Equilibrium Point

**FIGURE 3:**
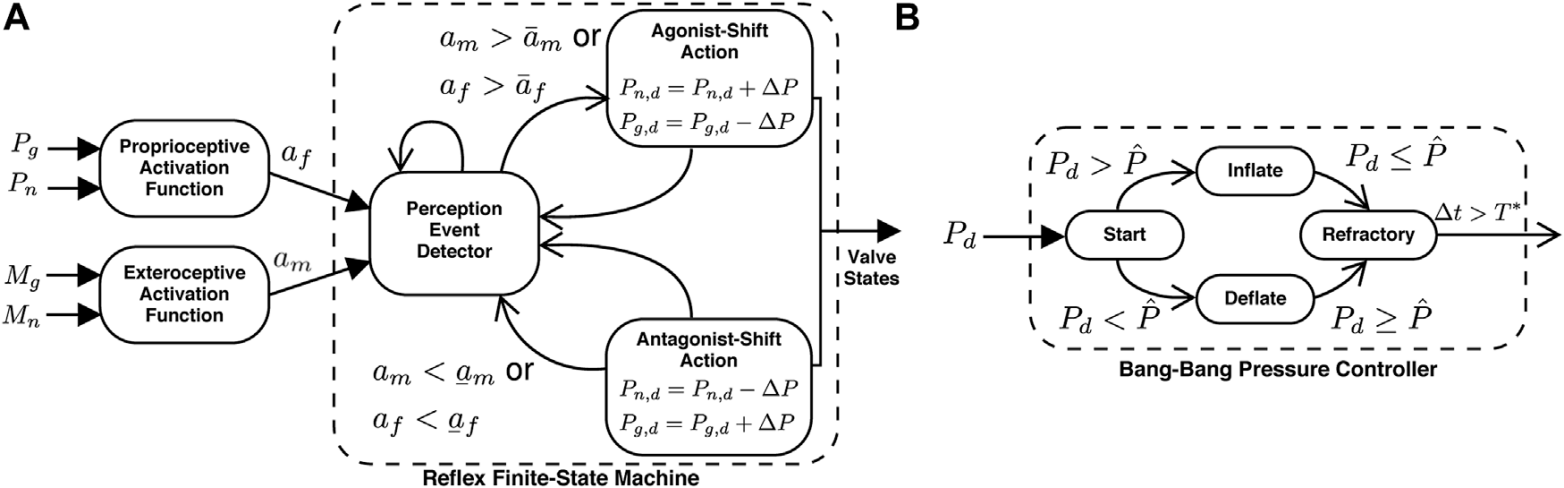
| Schematic representation of the equilibrium-point controller consists of the reflex and bang–bang finite-state machines. **(A)** The reflex finite-state machine triggers the appropriate action based on the outputs of the activation functions. The proprioceptive inputs include the internal pressure of the actuators (*P*_*g*_ and *P*_*n*_) and are processed by the proprioceptive activation function (Eq. 1). The exteroceptive inputs include the biological muscle sEMG signals (*M*_*g*_ and *M*_*n*_), which are processed with the exteroceptive activation function (Eq. 2). The reflex finite-state machine consists of the perception-state (event detector) and the two possible action states (either agonist- or antagonist-shift). The action states, triggered *via* threshold crossings, set the desired pressures which are then achieved *via* the bang–bang controller. Once an action is completed, the robot returns to the perception state. **(B)** The bang–bang pressure controller sets the valve configurations. If the internal pressure of the actuator $\hat{P}$ (subscripts dropped for convenience) is less than the desired pressure *P*_*d*_, then the actuator is inflated. If the $\hat{P}$ is greater than the desired pressure *P*_*d*_, the actuator is deflated. After the internal pressure has been achieved, there is a refractory period T* before returning to the perception state.

In order to provide physical assistance, the equilibrium point of the robot must be shifted congruent with the user’s intention and ideally lead the user’s motion toward their new equilibrium point, which is unknown to the robot. Therefore, when the user’s intent is detected, the robot’s equilibrium point jumps to a new position in the same direction as the user’s intention and, in this way, provides physical assistance. To accomplish this, the robot controller takes the form of a finite-state machine with two states: the perception state and the action state. During the perception state, the robot continuously monitors the sensors. The transition from the perception state to the action state is triggered only if a threshold condition is detected. Once in the action state, the robot completes a predefined action before returning to the perception state. We use the term reflex (e.g., an automatic response to stimuli) to describe the transition from the perception state to the action state and back to the perception state. We use two types of reflexes: the F-Reflex, which responds to the user’s intentions detected *via* physical human–robot interactions (e.g., proprioception), and the M-Reflex, which responds to the user’s intentions detected *via* the sEMG neural interface (e.g., exteroception). The reflex finite-state machine is illustrated in Figure 3A.

##### 2.1.3.1 F-Reflexes

To detect physical interaction between the user and the wearable robot, the internal pressure signals are processed through the proprioceptive activation function:

$$a_{f}\left(t\right)=h_{HP}\left(t\right)*\left(\left(P_{g,0}-{\hat{P}}_{g}\left(t\right)\right)-\left(P_{n,0}-{\hat{P}}_{n}\left(t\right)\right)\right),$$ (1)

where *P*_*i*,0_ are the nominal pressures of the agonist/antagonist (subscript *i* denotes either actuator) and ${\hat{P}}_{i}\left(t\right)$ are the current estimated pressures of the actuators computed as the low-pass filtered output of the pressure sensors with cutoff *f*_sens_, * is the time-domain convolution operator, *h*_*HP*_(*t*) is the impulse response of a high-pass filter with cutoff *f*_*HP*_. The purpose of Eq. 1 is to detect high-frequency variations in the internal pressure of the actuators which corresponds to physical interactions between the human user and the robot. The sign of Eq. 1 determines the direction of physical interaction: if *a*_*f*_(*t*) > 0, then ${\hat{P}}_{g}\left(t\right)$ has decreased below the nominal *P*_*g*,0_ while ${\hat{P}}_{n}\left(t\right)$ has increased above the nominal *P*_*n*,0_ corresponding to the shortening of agonist and lengthening of the antagonist pneumatic actuators. The opposite is true if *a*_*f*_(*t*) < 0: lengthening of agonist and shortening of the antagonist actuator. Importantly, the high-pass filter in Eq. 1, which has DC-blocking characteristics, produces a bias-corrected comparative signal allowing the activation function to be tuned to detect a minimum rate of change of the internal pressures so that low-frequency variations due to bladder leakages or slow user movements do not trigger reflexes.

##### 2.1.3.2 M-Reflexes

In order to detect volitional movements of the user, the exteroceptive activation function takes the following form:

$$a_{m}\left(t\right)={\hat{M}}_{g}-{\hat{M}}_{n},$$ (2)

where ${\hat{M}}_{i}$ are the rectified, low-pass filtered, and normalized sEMG signals from each muscle. Note that if *a*_*m*_ is positive, the agonist is more active and, therefore, motion is in the agonist direction. The opposite is true if *a*_*m*_ is negative. In contrast to direct proportional sEMG control, where the actuator is activated in proportion to the underlying muscle activation (Hogan, 1976; Kao et al., 2010; Fougner et al., 2012; Lenzi et al., 2012; Lince et al., 2017), Eq. 2 is more aligned with correlating the muscle pair (agonist–antagonist) together to a joint’s kinematic configuration (Iimura et al., 2011; Ariga et al., 2012a; Ariga et al., 2012b; Hirai et al., 2015).

##### 2.1.3.3 Agonist- and Antagonist-Shift Actions

The reflexes are initiated when a threshold crossing of either activation function is detected, and therefore, the perception event detector in Figure 3 continuously evaluates for a potential crossing:

$$\left\{\begin{array}{c} \text{if}\;a_{m}>{\bar{a}}_{m}\text{ or }a_{f}>{\underline{a}}_{f}\;\to \text{ Agonist}-\text{Shift}\;\\ \text{if}\;a_{m}<{\bar{a}}_{m}\text{ or }a_{f}<{\underline{a}}_{f}\;\to \text{ Antagonist}-\text{Shift} \end{array}\right.$$ (3)

with the thresholds parameterized by ${\bar{a}}_{i}$ and ${\underline{a}}_{i}$ for each activation function. The first step in the action sequence is to shift the desired internal pressures of each muscle:

$$P_{g,d}=\left\{\begin{array}{c} P_{g,d}+\Delta P\;\text{if Agonist}-\text{Shift}\;\\ P_{g,d}-\Delta P\;\text{if Antagonist}-\text{Shift}\; \end{array}\right.\;P_{n,d}=\left\{\begin{array}{c} P_{n,d}-\Delta P\;\text{if Agonist}-\text{Shift}\;\\ P_{n,d}+\Delta P\;\text{if Antagonist}-\text{Shift} \end{array}\right.$$ (4)

where *P*_*n*,*d*_ is the desired internal pressure of the agonist, *P*_*n*,*d*_ is the desired internal pressure of the antagonist, and Δ*P* is the jump (shift) in the desired pressure and corresponds to the equilibrium-point shift. After the desired pressures have been set, a bang–bang controller, illustrated as a finite-state machine in Figure 3B, activates the valves to drive the internal pressures to the desired pressures. Note that each actuator utilizes independent bang–bang controllers. Before exiting the action state, the actuator has a refractory period T*, which allows the internal pressure signals to settle to avoid the possibility of chatter (e.g., rapidly alternating between reflexes). Immediately after the refractory period, the nominal pressure of each actuator are updated with the current pressure samples (e.g., *P*_*n*,0_ and *P*_*g*,0_ in Eq. 1), which ensures the activation function is initially smooth after the refractory period.

### 2.2 Soft Wearable Robot Design

#### 2.2.1 Overview

**FIGURE 4:**
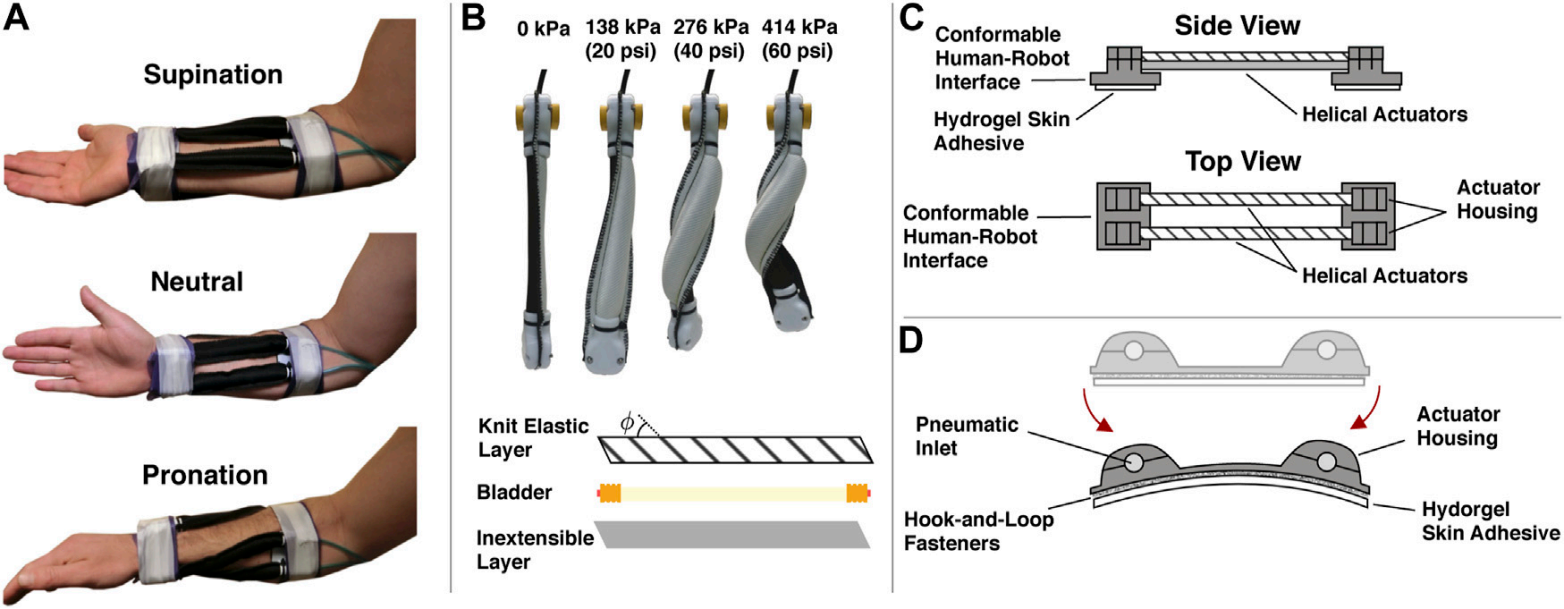
The soft wearable robot hardware characteristics. **(A)** Medial view of the soft robotic orthosis during supination, neutral, and pronation configurations. Two antagonistic robotic orthosis elements are worn by the user on either side of the forearm. The element on the dorsal side is configured to provide a pronating torque, while the element on the ventral side provides a supinating torque. Cohesive bandage (Coban) and medical tape secure the human–robotic interface to the human user. **(B)** Inflation sequence of a soft helical actuator and actuator construction details. A knit elastic fabric layer (with knit angle *ϕ*) and an inextensible fabric layer are sewn together with a latex bladder enclosed. **(C)** Side and top view schematics of a one soft wearable robot module. A pair of helical actuators are attached at both ends to conformable human–robot interfaces. The conformable human–robot interfaces include actuator housing sections that fasten and secure the actuators to the interfaces through cable ties (not shown). Removable hydrogel skin adhesives help attach the robotic orthosis to the human skin. **(D)** Conformable human–robot interface details. The conformable human–robot interface is designed to bend around and adhere to humans. Disposable hydrogel adhesives are attached *via* hook-and-loop fasteners to the underside of the human–robot interface and adhere to the user’s skin reducing the relative motion between the human and robot.

The soft wearable robot is fabricated from two actuator modules, each consisting of two parallel actuators. The modules are attached to the ventral and dorsal sides of the forearm and secured near the wrist and elbow. The ventral module provides motion in the supination direction, and the dorsal module provides motion in the pronation direction. A sequence of forearm motions illustrating the behavior of the device is provided in Figure 4A. The overall design used here is a variation of our previous work (Realmuto and Sanger, 2019). Three sizes of the soft wearable robot were fabricated (small, medium, and large) with lengths of 13, 16, and 19 cm, respectively, and the total on-body weight of each was approximately 100, 114, and 117 g, respectively. Based on previous empirical evaluations of the pneumatic helical actuators, we estimate they can produce approximately 1.5–2 Nm of assistive torque at maximum inflation used in this study (441 kPa (64 psi)) (Realmuto and Sanger, 2019).

#### 2.2.2 Actuator Design and Fabrication

The actuator fabrication process and inflation sequence are shown in Figure 4B. In the following, we briefly summarize the process. First, a pattern is cut to the desired actuator dimensions using a knit elastic band for the top layer (Heavy Stretch Knit Elastic Band, Cisone) and ballistic nylon for the bottom (1,050 Denier Coated Ballistic Nylon Fabric, Magna Fabrics). Note that the bottom layer is practically inextensible, while the knit elastic band has anisotropic stiffness characteristics and only stretches in the longitudinal direction. Therefore, prior to pattern cutting, the knit elastic band must be aligned with the chosen knit angle to produce helical motion. In this study, we use a knit angle of 45°. The two layers can be sewn together to form a sleeve. Next, the bladder is assembled. We use two push-to-connect tube fittings (adapter, 3/16 “Stem OD x 5/32” Tube OD, McMaster-Carr) inserted and glued into a latex rubber tubing (1/4“ ID, 5/16” OD, McMaster-Carr). Brass hose ferrules (0.38” Hose End ID, McMaster-Carr) are also crimped at the ends (before the glue dries) to seal the bladder. The bladder can then be inserted into the sleeve.

#### 2.2.3 Conformable Human–Robot Interface

Each end of the bladder-sleeve assembly is attached to the 3D printed human–robot interface *via* dedicated actuator housing and cable ties, as seen in the schematic representation of a single soft wearable robot module in Figure 4C. The human–robot interface is designed to conform to the user’s body and therefore consists of a thin layer of 3D printed material. The human–robot interface includes special housing protrusions for the actuators to fit into and, with a housing cap, become fixed to the interface, as shown in Figure 4D. A hydrogel skin adhesive (TENS electrodes, FITOP Store), attached to the underside of the human–robot interface, adheres to the skin of the user to minimize the relative skin-robot displacements. The hydrogel skin adhesives are repurposed skin electrodes commonly used during transcutaneous electrical nerve stimulation. After attaching both modules to the user’s arm, the wearable robot is secured to the body with self-adherent elastic wrap (Coban, 3 M) and medical tape (micropore cloth medical tape, 3 M).

#### 2.2.4 Control System

The equilibrium-point controller is implemented in digital hardware (Beaglebone Black) with a loop frequency of 1 kHz. The sEMG electrodes (DE–2.1 electrodes, Delsys) have a custom second stage amplifier to condition the signals prior to digitizing and are downsampled to 250 Hz due to limitations in the analog-to-digital converter. After digitizing, the signals are full-wave rectified and low-pass filtered with a first-order digital low-pass with a 3 Hz cutoff. The actuators of each module are connected together to form a single pneumatic unit with the pressure sensor arranged external to the wearable robot and connected to both pneumatic units through pneumatic tubing. Each pneumatic unit is connected to two solenoid valves (SY113-SMO-PM-F, SMC): one valve connected to an air reservoir (maintained at 860 kPa (125 psi)) and the other an exhaust valve. Digital signals from the Beaglebone Black activate each solenoid *via* externally powered Darlington transistors. The reflexes are implemented in software with the M-Reflex (EMG) always taking precedence over the F-Reflex (Interaction). Therefore, at any given time step, if the two activation signals (*a*_*m*_ and *a*_*f*_ in Eq. 3) are contradictory, the action triggered will follow that of the *a*_*m*_ trigger. During the experiments, the control system is placed on a table next to the participants.

### 2.3 Experimental Methods

#### 2.3.1 Participants

Seven children with clinically diagnosed cerebral palsy or an acquired static motor deficit between 8 and 22 years old (6 men, 1 woman; mean = 15 years old; standard deviation = 4 years) were recruited from the Pediatric Movement Disorders Clinic at Children’s Hospital of Los Angeles. The characteristics of each participant are collected in Table 1, including their rating on the Barry–Albright Dystonia (BAD) scale (Barry et al., 1999), which quantifies the severity of posturing and involuntary movements (higher values correspond to more severe motor impairments). A parent or guardian of each participant gave written informed consent, and all children gave verbal or written assent to participate, including US Health Information Portability and Accountability Act (HIPAA) authorization for the use of medical and research records, according to the approval of University of Southern California’s Institutional Review Board^1^. Four other children initially enrolled in the study were excluded. One of the excluded participants could not complete the tracking task due to bradykinesia; one participant elected to stop part way through the experiments; and two participants could not activate the wearable robot with their sEMG signal.

**TABLE 1:** Study participant details.

Participant ID	Diagnosis	Upper extremity BAD score (out of 8)	Total BAD score (out of 32)	Age	Gender	Dominant hand	Orthosis size	F-Reflex threshold	M-Reflex threshold
P1	Glutaric acidemia type I	4	17	12	Male	Left	Medium	±0.025	±1
P2	Cerebral palsy	6	17	16	Male	Right	Large	±0.025	±1
P3	Cerebral palsy	3	6	22	Male	Left	Large	±0.025	±1
P4	Cerebral palsy	6	11	18	Male	Left	Medium	±0.025	±1
P5	ATP1A3 gene mutation	3	11	13	Male	Left	Medium	±0.025	±1
P6	Cerebral palsy	4	15	8	Female	Left	Small	±0.025	(−1, 4)
P7	Cerebral palsy	5	16	14	Male	Left	Large	±0.025	±1

#### 2.3.2 Haptic Environment and Tracking Task

**FIGURE 5:**
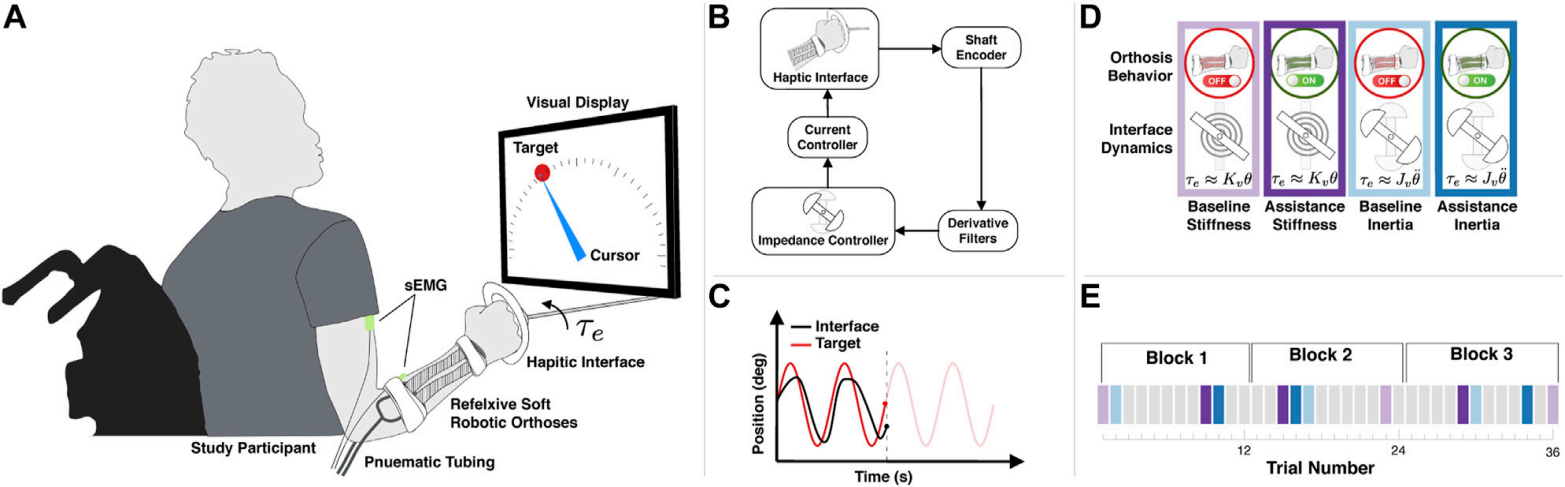
An overview of the experimental design. **(A)** The experiment consists of a participant with the soft wearable robot interacting with a one-degree-of-freedom rotational haptic interface. The total external torque *τ*_*e*_ depends on the haptic environment (Eq. 5). For each trial, the goal is to track the moving target with the cursor, directly controlled by the haptic interface. A display provides the participant with visual feedback of the target (red circle) and cursor (blue pointer) positions. The haptic interface supplies an external torque *τ*_*e*_ determined by either Stiffness-Dominate or Inertia-Dominate dynamics. **(B)** The haptic interface uses an impedance controller with an optical encoder on the shaft, providing the requisite feedback to render the appropriate torque at the interface. **(C)** A sample trial illustrating typical target (red) and interface (black) trajectories. In this study, the target trajectory consists of 15 cycles of a 0.2 Hz sinusoid with the amplitude spanning ± 80°. **(D)** Visual description of the four experimental conditions analyzed. **(E)** Example of one experimental timeline. During each block, the participant completed 12 distinct trials, four of which consisted of one of the experiment conditions analyzed in this study and described in **(D)** or an alternative task depicted as grey boxes and not included in the data analysis.

The experimental setup is illustrated in Figure 5A. The setup consisted of the participant physically grasping the handle of a one-degree-of-freedom rotational haptic device with an adjacent computer screen that displayed the desired tracking trajectory and the participant’s current position. The haptic device consisted of a brushless DC motor and gearhead (EC 45 Flat, 42.9 mm, 30 W brushless motor, and 36:1 Planetary Gearhead, Maxon) attached to a shaft with a handle for the user to interact with. The motor assembly was securely fixed to a table. An encoder at the shaft supplied the requisite angular feedback (AMT20 Series, CUI Devices). The motor behavior was directly controlled by the Beaglebone Black hardware, with a 1 kHz loop frequency, *via* an external motor current control board (ESCON 36/3 EC, 4-Q Servocontroller for EC motors, Maxon) that took as input an analog set point for the desired motor current. In order to render the dynamic environments, an impedance controller was used to produce the interaction torques (Hogan and Buerger, 2018). The control law for the impedance controller took the form:

$$i_{m}=-J_{v}{\ddot{\theta }}_{p}-K_{v}\theta ,$$ (5)

where *i*_*m*_ is the motor command current, ${\ddot{\theta }}_{p}$ is an estimate of the angular acceleration of the interface shaft, *θ* is the angular position of the shaft, *J*_*v*_ is the virtual inertia constant, and *K*_*v*_ is the virtual stiffness constant. The acceleration estimate ${\ddot{\theta }}_{p}$ is computed *via* a series of two digital impulse response filters with a cutoff frequency 5 Hz. The total external torque felt by the human at the handle interface is approximately equal to the product of the desired current, motor torque constant, and the gear ratio:

$$\tau_{e}=Rk_{\tau }i_{m},$$ (6)

with the torque constant *k*_*τ*_ = 0.0255 Nm/A and the gear ration *R* = 36. Eq. 6 ignores the friction in the shaft and motor inertia; therefore, *τ*_*e*_ is just an estimate. A schematic of the impedance controller can be seen in Figure 4B. The goal of the participant was to track 15 cycles of 0.2 Hz sinusoid trajectory, which ranges from −80° to 80°, as shown in Figure 5C.

#### 2.3.3 Experimental Conditions

In this study, we analyze the behavior of the participants in two specific haptic environments (Inertia-Dominant and Stiffness-Dominant environments) under two specific conditions (the baseline condition, where the robot is inactive, and the assistance condition, where the robot is activate. Figure 5D provides a summary of the experimental conditions. During the Inertia-Dominant environment, the virtual impedance constants in Eq. 5 were chosen as *J*_*v*_ = 4 and *K*_*v*_ = 0. During the Stiffness-Dominant environment, the virtual impedance constants in Eq. 5 were chosen as *J*_*v*_ = 0 and *K*_*v*_ = 0.03. The impedance constants were chosen through trial and error, and particular care was used in choosing the stiffness impedance constants in order not to cause instabilities during the human interactions.

#### 2.3.4 Equilibrium-Point Controller Settings

The nominal parameters of the equilibrium-point controller were selected iteratively, through trial and error, to produce stable human–robot interactions. The primary challenge is that if the transients do not settle following an equilibrium shift, the F-Reflex activation function (Eq. 1) could rapidly spike, causing another reflex. Therefore, we first tuned the F-Reflex parameters to eliminate this possibility. First, we selected the high pass filter cutoff *f*_*HP*_ such that signal bias from bladder leakage and slow user motions were removed. Starting with a small jump parameter Δ*P*, a large refractory period T*, and large thresholds for stable interactions (no self-induced reflexes), we next iteratively increased the jump parameter Δ*P* and decreased the refractory period T* until satisfactory responsiveness. The jump parameter Δ*P* effectively determines the amount of assistance the human user receives at each equilibrium shift. If the parameter is too small, the assistance will lag the user. If the parameter is too large, the assistance could overshoot the user and induce human reflexes. We selected the jump parameter Δ*P* such that assistance was clearly felt but did not induce a reflexive response from the human. The nominal thresholds (which could be tuned to each individual) were next fine-tuned to provide a higher degree of responsiveness. The nominal M-Reflex threshold parameters were subsequently chosen to ensure volitional control through the sEMG interface. The M-Reflex activation function (Eq. 2), which uses the sEMG signal envelops (rectified low-pass filtered, see Section 2.2.4), did not require the same considerations, and the most important parameters were the M-Reflex thresholds. We arrived at nominal thresholds (which could be tuned to each individual) by starting with large thresholds and iteratively decreasing them until satisfactory volitional control was possible. The parameters were selected as follows: the jump parameter was set to Δ*P* = 55 kPa (8 psi); the refractory period was chosen as T* = 150 milliseconds; the initial desired pressure *P*_*n*,*d*_ = *P*_*g*,*d*_ = 276 kPa (40 psi); the cutoff of the high-pass, which was implemented as a first-order digital infinite impulse response filter, was chosen as *f*_*HP*_ = 223 Hz; and the cutoff frequency of the low-pass filter for pressure sensors, also implemented as finite infinite response filters, was set to *f*_*sens*_ = 3 Hz. The maximum internal pressure of the actuators was capped to *P*_max_ = 441 kPa (64 psi) and the minimum *P*_min_ = 103 kPa (15 psi). The nominal F-Reflex thresholds ( ${\bar{a}}_{f}$ and ${\underline{a}}_{f}$ ) were chosen as ± 0.025, and the nominal M-Reflex thresholds ( ${\bar{a}}_{m}$ and ${\underline{a}}_{m}$ ) were chosen as ± 1.

#### 2.3.5 Protocol

After the participant completed the informed consent process, the two electrodes were placed on the bicep and pronator teres, which are known to correlate with supination and pronation, respectively (Bader et al., 2018). The ventral (supinator) pair of actuators were arranged alongside and/or slightly on top of the low-profile surface EMG electrode located over the pronator teres. We used medical tape in conjunction with the standard electrode adhesive to minimize possible electrode migration. The conformable human–robot interface, which had the hydrogel pads attached to the underside, was always placed proximal to the electrodes and therefore did not interfere. Our preliminary exploratory experiments did not indicate any substantial effect on signal quality or the ability of the user to activate the wearable robot when the supinator pair of actuators were inflated, and therefore, we do not believe there is a strong effect of the supinator actuator compromising the sEMG signal quality. After the wearable robot was attached to the participant, they were given a few minutes to become acclimated to the device, including the equilibrium-point controller. If the participant could not trigger the M-Reflex or F-Reflex, the nominal threshold parameters were adjusted until they could. For all but one participant (P6), the nominal parameters were sufficient. The reflex parameters are recorded in Table 1. The experiment was divided into blocks, with each block consisting of a series of 12 distinct tasks. In this study, we only compare and analyze four tasks, as previously elaborated and summarized in Figure 5D. Six of the other tasks were related to a ballistic reaching task, and the other two had an alternative setting of the soft wearable robot (M-Reflex only), both of which we do not analyze in this study. During the first experimental block, the experimental conditions were presented in the following order: baseline/Stiffness-Dominate, baseline/Inertia-Dominate, assistance/Stiffness-Dominate, and assistance/Inertia-Dominate. For each of the subsequent experimental blocks, the conditions were presented in random order. An example of one of the experimental timelines is illustrated in Figure 5E. Between each trial (task), the participant was given up to a 1 min break but was allowed to proceed if they wanted to. After each block, the participant was given a 5-minute break. Each participant was required to complete three experimental blocks, after which they were asked to complete a fourth. Three participants completed four blocks, while the other four completed three blocks.

#### 2.3.6 Demonstration Videos

In order to highlight and clarify the equilibrium-point control method, a video of the F-Reflex response in real time during a bench-top experiment is included as shown in Supplementary Video S1, and the data are also shown in Supplementary Figure S1. The demonstration video shows the wearable robot responding to external interactions while mounted to a bench-top with corresponding real-time data, including traces of the activation signal, the internal pressures, the desired pressures, and the solenoid valve states. In addition, a video of one of the experimental trials (Participant P7, Block 1, Trial 9, assistance/Stiffness-Dominant condition) is included as shown in Supplementary Video S2, and the data are also shown in Supplementary Figure S2. These demonstrations provide concrete examples of the underlying activation signals and subsequent reflexes during the interaction.

#### 2.3.7 Code

All codes used in the experiment, including those for the equilibrium-point controller and the haptic interface, are open-source and available for download^2^.

## 3 Results

### 3.1 Inertia-Dominant Haptic Environment

**FIGURE 6:**
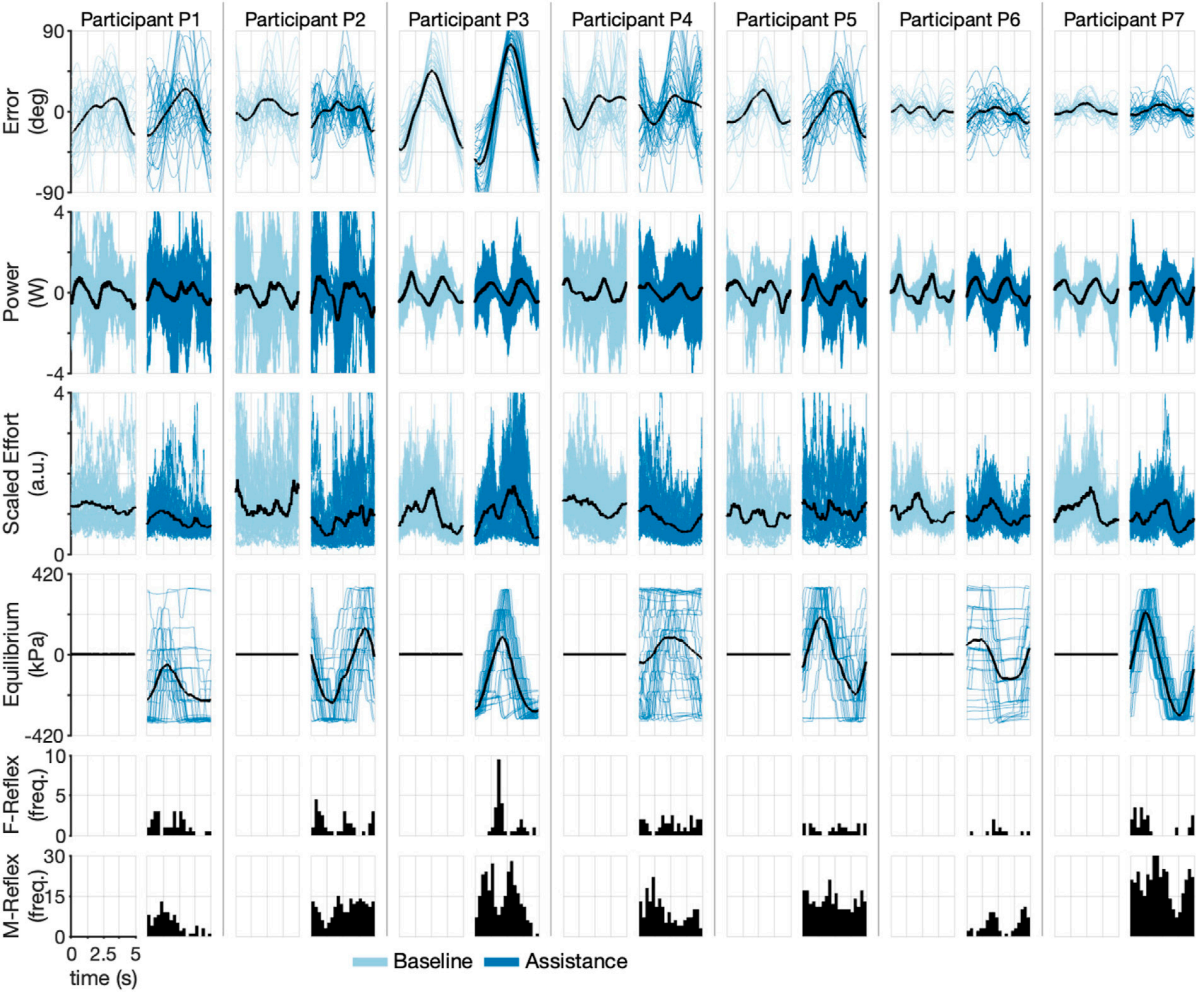
Time-domain experimental results during the Inertia-Dominant environment. Each pair of columns (one for baseline and one for assistance conditions) represents a single participant. Each row shows a different experimental variable. The dark line represents mean. Shaded lines are individual cycles. The last two rows show cycle time histograms (timing of each reflex within a cycle) of each type of triggered reflex (F-Reflex, M-Reflex).

To understand participant performance and the effect of robotic assistance, we segmented the data into individual cycles, discarding the first five cycles of each trial to reduce the initial transient effects and using the last 10 cycles per trial to find the point-wise mean over all cycles of each of the following outcomes: error, calculated as the difference between the desired and actual position of the haptic interface; interaction power, calculated as the product of the angular velocity of the haptic interface and the torque command of the haptic interface; effort, calculated as the sum of the linear envelop of the bicep and pronator teres sEMG signals, with each channel normalized by their respective ensemble peak values (Besomi et al., 2020); robot equilibrium point, calculated as the difference between the filtered versions of the internal pressures of each actuator; and reflex timing for both types of reflexes, which is the time the trigger activated within each cycle. We note that the equilibrium point of the robot is reported as the pressure differential between the antagonistic actuators because the true equilibrium is not easily measured due to the compliant nature of the soft actuators (see Figure 4B and Supplementary Video S1). As reported in this way, the maximum differential corresponds to maximal supination, the minimum differential corresponds to maximal pronation, and the zero differential corresponds to neutral equilibrium. The participants’ performances were highly variable, as seen in Figure 6, which shows the participant-specific outcomes for each cycle with their respective point-wise mean over all cycles. For example, while Participant P3 had peak mean tracking errors on the order of 45° during baseline and 90° during assistance, Participant P7 had a peak mean tracking error below 10° for both baseline and assistance. Similar participant-specific trends can be seen for each outcome, confirming the highly variable abilities of the participants.

To understand the ability of participants to activate reflexes, we calculated the average reflex per cycle, shown in Supplementary Figure S3. Participant P7 averaged the highest M-type reflex per cycle (9.9), followed by P5 (8.7), P3 (7.1), P2 (7.0), P1 (3.1), and P6 (2.8). The participants triggered the F-type reflex less frequently, with all participants except for P6 averaging approximately 1–2 per cycle and P6 averaging below one. We also calculated the equilibrium point of the robot for each cycle (fourth row of Figure 6). Because the underlying desired trajectory is a sinusoid, we would expect the robot’s equilibrium point to also be sinusoidal, as in Participants P2 and P7, and to a lesser degree P3 and P5. Participant P7 appears to have leveraged the robotic assistance best, with large decreases in effort corresponding to the maximum equilibrium points of the robot (compare rows three and four of Figure 6). A similar trend is seen for P2, who is right-handed and therefore has a robot equilibrium point that appears inverted relative to the other participants. These results confirm that all participants, to varying degrees, could modulate the equilibrium point of the robot.

**FIGURE 7:**
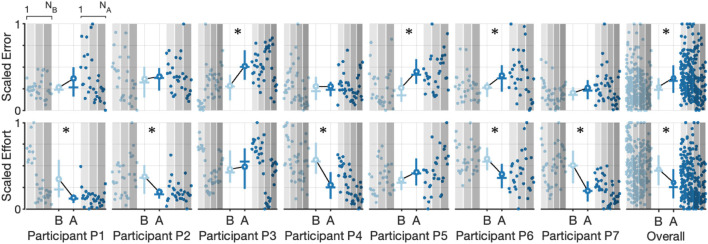
Scatter plots and corresponding interquartile range (Q3-Q1) shown as vertical lines, mean value (solid circle), median value (horizontal line), and the trend line for scaled error and scaled effort for each cycle during the Inertia-Dominate condition. Observations are plotted in order (1 to *N*_*B*_ for the baseline condition and 1 to *N*_*A*_ for the assistance condition). Increasing shaded regions denote each experimental block. Asterisks denote a significant (*p* ≤0.05 ) test decision for the null hypothesis that the data from both conditions are from the same distribution using a two-sample Kolmogorov–Smirnov test.

To further explore the participant-specific performances and the effect of robot assistance during the Inertia-Dominant environment, we used the cycle-segmented data to calculate the root mean square (RMS) error and the integral of effort, calculated with the trapezoid rule for each individual cycle, thereby generating a single observation for each cycle. We then rescale the data within each subject to between zero and one. The resulting scatter plot, with the mean, median, and interquartile ranges, is shown in Figure 7, which compares the baseline and assistance conditions. Each data point is plotted in order of observation, from 1 to *N*_B_ (number of baseline cycles) and from 1 to *N*_A_ (number of assistance cycles). The outcomes are correlated in time, which can be seen by comparing each cycle observation over subsequent experimental blocks (denoted as increasing shaded regions). Qualitatively, subjects seem to decrease effort with increasing cycle number.

Using the two-sample Kolmogorov–Smirnov test (Berger and Zhou, 2014), we tested the hypothesis that the undying distributions, for effort and error, differed between the baseline and assistance conditions (all distributions failed a normality test, one-sample Kolmogorov–Smirnov test with *p* < 0.05). We found that the test decision of five of the participants rejected the null hypothesis (*p* < 0.05) that data were from the same underlying distributions for the effort outcome, with all five subjects having mean effort during the assistance condition lower than the mean effort during the baseline condition (denoted with an asterisk in Figure 7). On the contrary, we found that the test decision of three of the participants rejected the null hypothesis (*p* < 0.05) that data were from the same underlying distributions for the error outcome, with all three subjects having a mean error during the assistance condition higher than the mean error during the baseline condition (denoted with an asterisk in Figure 7). Of the three participants observed to have significantly increased error between the baseline and assistance condition, only one (P6) was also observed to have significantly decreased effort. We repeated the analysis on the aggregate data (last column in Figure 7), with the results rejecting the null hypothesis for both the effort and error outcomes, providing mixed support to our central hypothesis that the robot will reduce user effort without increasing error. Summary statistics are presented in Supplementary Table S1.

### 3.2 Stiffness-Dominant Environment

**FIGURE 8:**
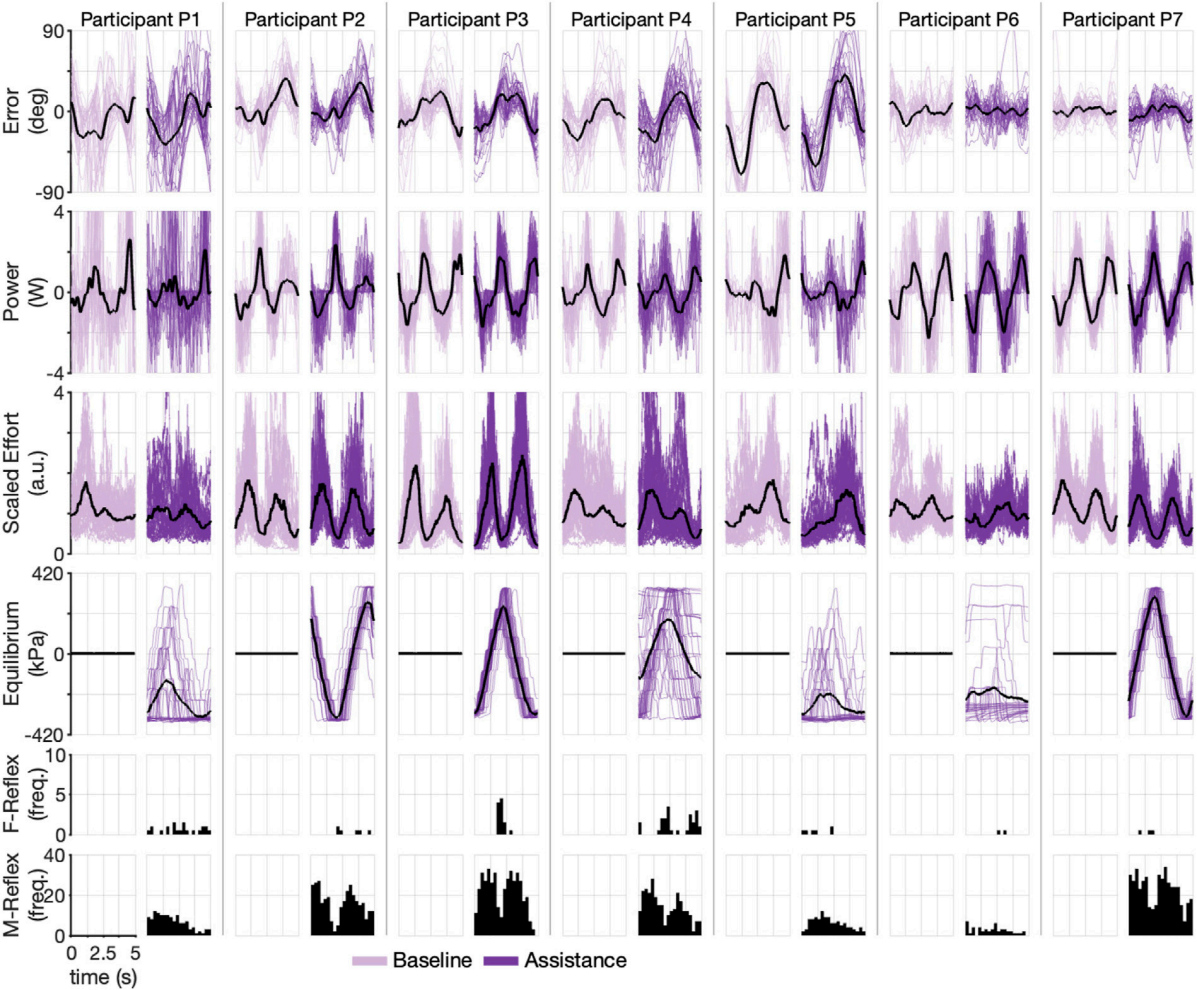
Time-domain experimental results during Stiffness-Dominant environment. Each pair of columns (one for baseline and one for assistance conditions) represents a single participant. Each row shows a different experimental variable. The dark line represents mean. Shaded lines are individual cycles. The last two rows show cycle time histograms (timing of each reflex within a cycle) of each type of triggered reflex (F-Reflex, M-Reflex).

To understand participant performance during the Stiffness-Dominant environment, we repeated the individual cycle analysis exactly as in the Inertia-Dominant case. The participants’ performances were again highly variable, as shown in Figure 8, presenting the participant-specific outcomes for each cycle with their respective point-wise cycle means. For example, while Participant P5 had peak mean tracking errors on the order of 60° during baseline and assistance conditions, Participant P7 again had peak mean tracking error below 10° for both baseline and assistance conditions. Similar participant-specific trends can be seen for each outcome, confirming the highly variable abilities of the participants.

To understand the ability of participants to activate reflexes during the Stiffness-Dominant environment, we calculated the average reflex per cycle, which is shown in Supplementary Figure S3. Participants P7 (11.4) and P2 (10.8) averaged the highest M-type reflex, followed by P3 (10.5), P4 (7.1), P1 (4.4), and P6 (1.6). The participants triggered the F-type reflex much less frequently, with most participants averaging less than one per cycle and P6 and P7 averaging nearly zero (∼0.07). In terms of the equilibrium point of the robot for each cycle (fourth row of Figure 8), the participants follow the same trend as the Inertia-Dominant environment, with P2, P3, and P7 producing clear sinusoids and the others having more variations. Participant P7 again appears to have leveraged the robotic assistance best, with large decreases in effort corresponding to the maximum equilibrium points of the robot (compare rows three and four of Figure 8). A similar trend is also seen for P2 (who is right-handed). These results confirm that the participants, to varying degrees, could modulate the equilibrium point of the robot during the Stiffness-Dominant condition.

The Stiffness-Dominant environment required more mechanical power during the task. This can be seen by comparing the interaction power between the two conditions (the second row in Figure 6 *versus* second row in Figure 8). Peak mean power during the Stiffness-Dominant environment was often above 2 W, which is substantially more than that during the Inertia-Dominant environment. The underlying effort signals are also qualitatively different between the two environments, suggesting that the motor strategies are tailored to the dynamic environment.

We further explored the participant-specific performances and the effect of robot assistance during the Stiffness-Dominant tracking environment using the identical analysis for the Inertia-Dominant environment, where we used segmented cycles to calculate the root mean square (RMS) error and the integral of effort per cycle. The resulting scatter plot, with the mean, median, and interquartile ranges, is shown in Figure 7, comparing the baseline and assistance conditions. Qualitatively, most subjects seem to decrease effort with increasing cycle number.

**FIGURE 9:**
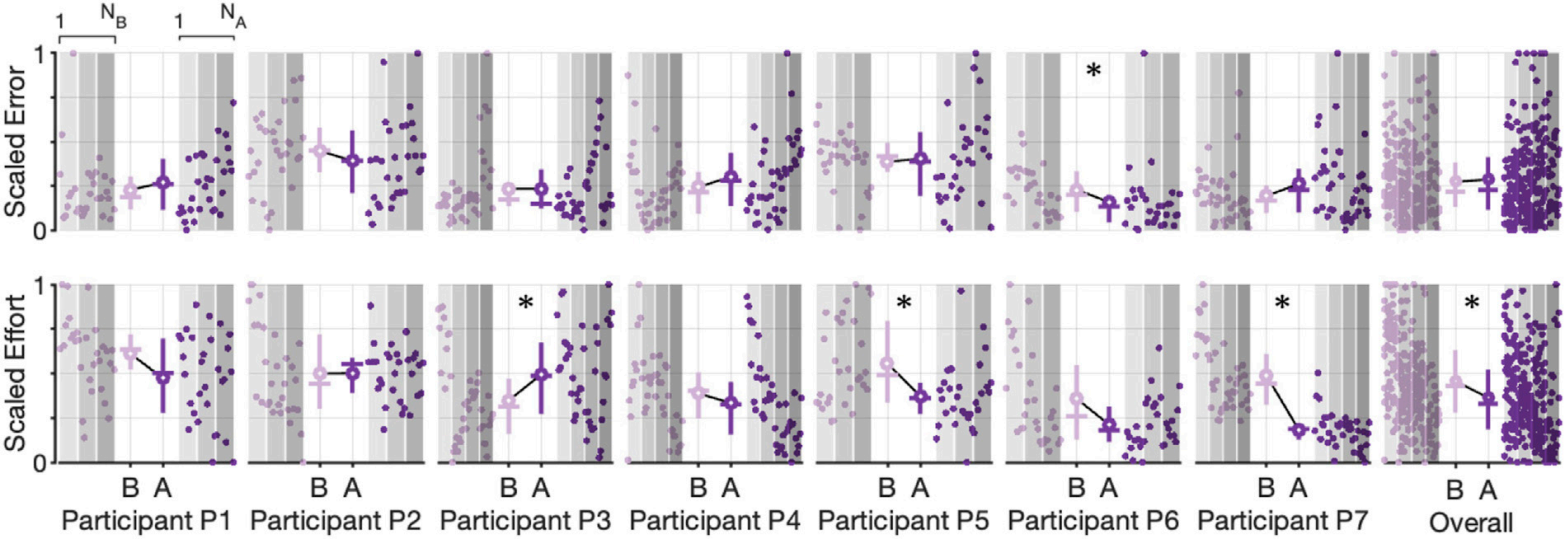
Scatter plots and corresponding interquartile range (Q3-Q1) shown as vertical lines, mean value (solid circle), median value (horizontal line), and trend line for scaled error and scaled effort for each cycle during the Stiffness-Dominate condition. Observations are plotted in order (1 to *N*_*B*_ for the baseline condition and 1 to *N*_*A*_ for the assistance condition). Increasing shaded regions denote each experimental block. Asterisks denote a significant (*p* ≤0.05 ) test decision for the null hypothesis that the data from both conditions are from the same distribution using a two-sample Kolmogorov–Smirnov test.

We again used the two-sample Kolmogorov–Smirnov test to test the hypothesis that the undying distributions, for effort and error, differed between the baseline and assistance conditions. We found that the test decisions of three participants rejected the null hypothesis (*p* < 0.05) that data were from the same underlying distributions for the effort outcome, with two participants (P5 and P7) having mean effort during the assistance condition lower than the mean effort during the baseline condition and one participants (P3) having an increase in mean effort during the assistance condition (denoted with an asterisk in Figure 9). We found that the test decision of one participant rejected the null hypothesis (*p* < 0.05) that data were from the same underlying distributions for the error outcome, with the participant having a mean error during the assistance condition higher than the mean error during the baseline condition (denoted with an asterisk in Figure 9). We repeated the analysis on the aggregate data (last column in Figure 9, with the results rejecting the null hypothesis for only the effort outcomes, supporting our central hypothesis that the robot will reduce user effort without increasing effort. Summary statistics are presented in Supplementary Table S2.

### 3.3 Linear Mixed-Effects Model

The dataset from this study is correlated within each participant, trial, and haptic environment. To account for these correlations, gain insights into the important predictors of user effort and error, and further test our hypotheses, we fit a linear mixed-effects model to the cycle level observations (Figures 7, 9). Our mixed-effects model took the form

$$E_{i,j}=\left(\beta_{0}+\alpha_{0,i,j}\right)+\beta_{1}P_{i,j}+\left(\beta_{2}+\alpha_{2,i,j}\right)C_{i,j}+\left(\beta_{3}+\alpha_{3,i,j}\right)t_{i,j}+\epsilon_{i,j},$$ (7)

where *E*_*i*,*j*_ is the integral of effort during each cycle for participant *i* during haptic environment *j*, predictor *P*_*i*,*j*_ corresponds to the RMS interaction power during each cycle, predictor *C*_*i*,*j*_ is the (categorical) assist condition (baseline or assistance), predictor *t*_*i*,*j*_ is the cycle number scaled to between 0 (first cycle on first trial of all experimental trials, including those not analyzed in the study) and 1 (last cycle on last trial of all experimental trials, see Figure 5E), *ϵ*_*i*,*j*_ is the random errors term, *β*′s are the fixed effects, and *α*′s are the random effects. The random effects are assumed independent and normally distributed, that is, $\alpha_{0,i,j}∼N\left(0,\sigma_{0}^{2}\right)$ , $\alpha_{2,i,j}∼N\left(0,\sigma_{2}^{2}\right)$ , and $\alpha_{3,i,j}∼N\left(0,\sigma_{3}^{2}\right)$ , where *σ* ^2^’s are the components of variation for each random effect. The residual is also assumed normally distributed, that is, $\epsilon_{i,j}∼N\left(0,\sigma_{\epsilon }^{2}\right)$ , with $\sigma_{\epsilon }^{2}$ the residual component of variation. The model in Eq. 7 represents a random intercept and random slope model with uncorrelated random effects and was chosen through a model selection process where candidate models were fit using the experimental data and evaluated based on the Bayesian information criterion (BIC). A summary of the candidate models is provided in Supplementary Figure S4. All models were fit using maximum-likelihood with the fitlme method in MATLAB (MathWorks).

**FIGURE 10:**
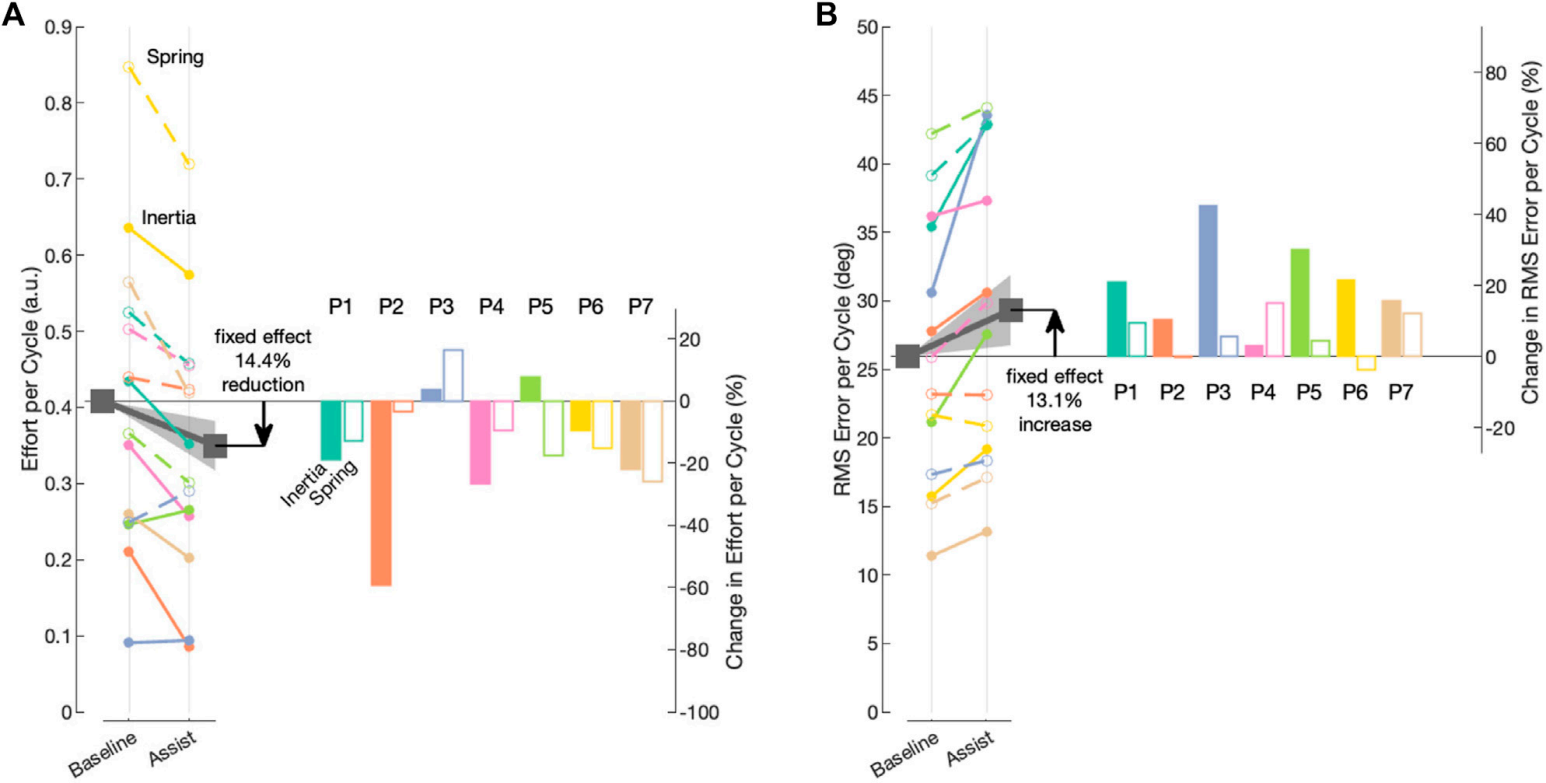
Visual representation of the fixed and random effects from the fitted mixed-effects models for the intercept and assist independent variables. Fixed effects are denoted by gray squares, and the trend line with the shaded region represents 95% confidence intervals. Random-effects trend lines for each participant are denoted by different colors, and random effects for the dynamic (Stiffness- or Inertia-Dominate) are denoted by open and closed circles, respectively. Corresponding bar plots depict the subject-specific and dynamic-specific percent change in outcome (e.g., random effects). **(A)** Model for effort as the outcome variable. **(B)** Model for error as the outcome variable.

A summary of the fitted fixed-effects parameters and the random-effects variance parameters are provided in Table 2. For the purpose of our hypothesis, the most important predictor is the categorical assist, which was found to have a coefficient of *β*_2_ = −0.059 (95% confidence intervals from −0.092 to −0.027 *p* ≈ 3.8e-4), supporting part of our hypothesis that the robot assistance will decrease user effort. To visualize the fitted model, Supplementary Figure S5 presents the partial residual plots, which show each dependent variable corrected for all independent variables except the one of interest (Larsen and McCleary, 1972). To visualize the fixed and random effects of the intercept (*β*_0_ + *α*_0,*i*,*j*_) and assist predictor (*β*_2_ + *α*_2,*i*,*j*_), we plotted the trend lines from each intercept during the baseline condition to the fitted assist condition for all subjects in Figure 10A. The results show that the assistance from the wearable robot reduced effort in both haptic environments for all but two participants (P3 & P5), and the fixed effect corresponds to a 14% decrease in effort with a 95% confidence interval from a 6.5% to 22.4% decrease in the effort.

**TABLE 2:** Mixed-effects model for user effort.

Model: effort ∼ 1 + power + assist + cycle number + (1∣ID:dynamic) + (−1 + assist∣ID:dynamic) + (−1 + cycle number∣ID:dynamic)							
Number of observations	960	AIC	−1,697.7
Fixed-effects coefficients	4	BIC	−1,658.8
Random-effects coefficients	42	Log-likelihood	856.87
Covariance parameters	4	Deviance	−1,713.7
Fixed-effects coefficients (95% CIs)							
Name	Estimate	SE	t statistic	df	*p*-value	Lower	Upper
Intercept	0.40871	0.053393	7.6548	956	4.7e − 14	0.30393	0.51349
Power	0.084505	0.01185	7.131	956	2.0e − 12	0.061249	0.10776
Assist	−0.05902	0.016569	−3.562	956	0.00039	−0.091537	−0.026504
Cycle number	−0.083379	0.033781	−2.4682	956	0.014	−0.14967	−0.017085
Random-effects variance parameters (95% CIs)							
Name	Levels	Type	Estimate	Lower	Upper	VPC^1^
Intercept	14	SD	0.19252	0.13215	0.28048	0.5829
Assist	14	SD	0.057724	0.037737	0.088296	0.0524
Cycle number	14	SD	0.12141	0.081131	0.18169	0.2318
Error	NA	Residual SD	0.091925	0.087811	0.096231	NA

^1^VPC, variance partition coefficient calculated as shown in Eq. 8 with the estimated (mean) random-effects variance parameters.

Model formula is provided in Wilkinson notation (Wilkinson and Rogers, 1973). The grouping variable “ID” represents the participant and the grouping variable “dynamic” represents the haptic environment (Inertia- or Stiffness-Dominant).

To assess the random effect contributions to the total variance of the outcome, we calculated the variance partition coefficient (VPC) for each of the random effects (Monsalves et al., 2020). The VPC indicates the proportion of total variance that is accounted for by between-group variation, that is, the random effects clustered by participant and dynamic environment (the *i*, *j* subscripts in Eq. 7). The VPC for random-effect *k* was calculated as

$${\text{VPC}}_{k}=\frac{\sigma_{k}^{2}}{\sigma_{0}^{2}+\sigma_{2}^{2}+\sigma_{3}^{2}+\sigma_{\epsilon }^{2}}.$$ (8)

The calculated VPCs show that a large part of the total variance is explained by the participant/environment variance of the intercept random effects (*σ*_0_, VPC_0_ = 0.5829) and the cycle time random effects (*σ*_3_, VPC_3_ = 0.2318), while a smaller portion is explained by the variance of the assist random effects (*σ*_2_, VPC_2_ = 0.0524).

To analyze the effect of the robot assistance on error, we used the same linear mixed-effects model structure in Eq. 7 with the RMS cycle errors as the outcome variable. A summary of the fitted fixed-effects parameters and the random-effects variance parameters is provided in Table 3. To visualize the fitted model, Supplementary Figure S6 shows the partial residual plots, which show each dependent variable corrected for all independent variables except for the one of interest. For the purpose of our hypothesis, the most important predictor is the categorical assistance condition, which was found as *β*_2_ = 3.4026 (95% confidence intervals from 0.83 to 5.98 *p* ≈ 0.01), not supporting our hypothesis that the robot assistance will not increase error. To visualize the fixed and random effects of the intercept and condition independent variables for the error outcome, we plotted the trend lines from the intercept during the baseline condition to the fitted assistance condition for all subjects in Figure 10B. The results show that the assistance from the wearable robot increased error in both conditions for all but two participants (P2 and P6), both of which had a decrease in error during the Stiffness-Dominant environment. The fixed effect corresponds to a 13% increase in error with a 95% confidence interval from a 3.2% to 23.1% increase in error.

**TABLE 3:** Mixed-effects model for user error.

Model: error ∼ 1 + power + assist + cycle number + (1∣ID:dynamic) + (−1 + assist∣ID:dynamic) + (−1 + cycle number∣ID:dynamic)							
Number of observations	960	AIC	7433.8
Fixed-effects coefficients	4	BIC	7472.7
Random-effects coefficients	42	Log-likelihood	−3708.9
Covariance parameters	4	Deviance	7417.8
Fixed-effects coefficients (95% CIs)							
Name	Estimate	SE	t statistic	df	*p*-value	Lower	Upper
Intercept	25.918	3.0671	8.4504	956	1.0722e − 16	19.899	31.937
Power	−2.4973	1.3869	−1.8006	956	0.072085	−5.219	0.22451
Assist	3.4026	1.3108	2.5958	956	0.0095817	0.83021	5.975
Cycle number	−0.62148	2.1642	−0.28716	956	0.77405	−4.8687	3.6257
Random-effects variance parameters (95% CIs)							
Name	Levels	Type	Estimate	Lower	Upper	VPC^1^
Intercept	14	SD	9.617	6.4639	14.308	0.3326
Assist	14	SD	4.0992	2.4414	6.8828	0.0604
Cycle number	14	SD	6.9348	4.2439	11.332	0.1729
Error	NA	Residual SD	10.986	10.495	11.5	NA

^1^VPC, variance partition coefficient calculated as shown in Eq. 8 with the estimated (mean) random-effects variance parameters.

Model formula is provided in Wilkinson notation (Wilkinson and Rogers, 1973). The grouping variable “ID” represents the participant and the grouping variable “dynamic” represents the haptic environment (Inertia- or Stiffness-Dominant).

To assess the random effect contributions to the total variance of the error outcome, we again calculated the variance partition coefficient (VPC) for each of the random effects (Monsalves et al., 2020). The calculated VPCs show that some part of the total variance is explained by the participant/environment variance of the intercept random effects (*σ*_0_, VPC_0_ = 0.3326) and the cycle time random effects (*σ*_3_, VPC_3_ = 0.1729), while a small portion is explained by the variance of the assist random effects (*σ*_2_, VPC_2_ = 0.0604).

**FIGURE 11:**
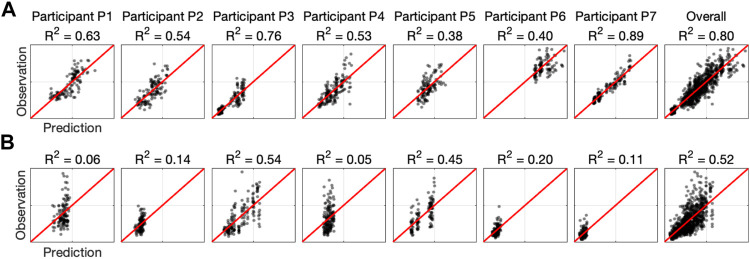
Summary of linear mixed-effects model fits. Each plot shows the observations *versus* predictions for each participant and overall. The red line denotes the perfect model. **(A)** Model with effort as the outcome variable. **(B)** Model with error as the outcome variable.

The model fits provide a different level of predictive power. In order to visualize this, the participant-specific and aggregate model fits are shown in Figure 11, which plots the observations versus the predictions for both outcome variables (effort and error). If the fit was perfect, the data points would fall on the *y* = *x* line. The error model is substantially less powerful, as evident by the lower R-squared measure.

In summary, according to our linear mixed-effects models, for six out of the seven participants, the robot assistance reduced their required effort in at least one of the haptic environments, and for five of the participants, the assistance reduced effort in both haptic environments. On the contrary, the robot assistance increased the tracking error in all participants during the Inertia-Dominant environment and in all but two during the Stiffness-Dominant environment.

## 4 Discussion

Soft pneumatic-based actuators are difficult to control due to inherent compliance and nonlinear effects. To circumvent these difficulties, we proposed a bio-inspired control approach to leverage the passive mechanical properties of the actuators together with their proprioceptive capabilities. By arranging them antagonistically, the actuators produced stable equilibria without direct feedback control. Using proprioception and with human input in the form of sEMG, our wearable robot shifted its equilibrium congruent with the user’s intentions, thereby providing physical assistance. The method is the first step in an impedance modulation framework.

Our main objective was to evaluate the ability of the soft wearable robot with equilibrium-point control to provide physical assistance to forearm rotations in pediatric users who have movement disorders. We evaluated the participants through a tracking task in two different haptic environments designed to require different movement strategies, confirmed by the markedly different effort signals between the two tasks (the third row in Figures 6, 8). Though, the proposed control method achieved volitional assistance for most users in both dynamic environments.

The two linear mixed-effects models, one with effort as the outcome and the other with error as the outcome, provide insights into the primary factors associated with participant performance. The models captured the highly variable capabilities of the participant population within each haptic environment through the random-effects intercept term, which explained a large portion of the variance in the models (VPC of 58% and 33% for the effort and error models, respectively). The cycle number predictor showed that the participants expended less effort over time, with the coefficient *β*_3_ = −0.083 (95% confidence intervals from −0.15 to 0.02 *p* = 0.014), while the same predictor in the error model was not significant. This suggests a learning effect, where the participants adapted their motor strategies resulting in less effort with increasing time while not significantly altering the tasks error. The power predictor, which did not have an associated random effect, shows an effort increase with increasing mechanical interaction (*β*_1_ = 0.085 (95% confidence intervals from 0.061 to 0.11 *p* < 0.01), while not reaching significance in the error model. The assist predictor did not explain a substantial portion of the variance in either model (VPC of 5% and 6% for the effort and error models, respectively), suggesting that the assist random effects had a relatively small associated variance and therefore the assistance from the wearable robot was consistent in reducing effort and increasing error across all participants and the two haptic environments.

Our results show that the soft wearable robot with the equilibrium-point controller provided physical assistance to the pediatric participants. The integral of effort was reduced for most participants in both dynamic manipulation tasks. However, the soft wearable robot also increased the resulting RMS error. Because the model fit is stronger for the effort outcome (Figure 11), we have more confidence in the reported effort metrics than the error outcome. However, there is clear evidence that wearable robots increase errors to some degree. We believe the increase in error can be explained by the relatively coarse assistance (equilibrium-point) profiles (see the fourth row in Figures 6, 8). Because the jump parameter was chosen as a fixed value (Δ*P* = 55 kPa) and the minimum internal pressure was set to *P*_min_ = 103 kPa, the equilibrium point could only take on seven unique equilibria, which would not be enough to produce fine motor control. The effect is clearer in the Inertia-Dominant condition (Figure 6) due to the subtle motor control required, including breaking and accelerating when changing direction.

All participants activated the M-Reflex (sEMG) at higher rates compared to the F-Reflex (Interaction), as shown in the reflex histograms (last two rows) in Figures 6, 8. This is expected because sEMG signals lead motion in phase and therefore provide a superior prediction of the user’s volitional movement. Based on our results, the F-Reflex was triggered more often during the Inertia-Dominant environment, which requires substantial acceleration/deceleration when switching directions and also very little joint torque (therefore muscle activation) during the constant velocity portions of the trajectory (i.e., between the crests/troughs of the sine signal). This may explain why, for many participants, the F-Reflex was triggered most often early, in the middle, and late during the cycle (see P1, P2, P3, P4, and P7 histograms in Figure 6). These instances corresponded to when the desired trajectory was approximately constant velocity. Combined with the momentum of the Inertia-Dominant interface, little muscle activation was necessary to keep the interface moving, confirming that the F-Reflex could provide assistance during these portions of the trajectory in the absence of strong muscle activations.

Based on the results and the observations obtained in this study, we believe that the performance of our soft wearable robot could be further improved. The role and individual benefit of each reflex (M-Reflex and F-Reflex) require further investigation. In this study, the M-Reflex (EMG) always takes precedence over the F-Reflex (interaction). However, there are conceivable scenarios where the two corresponding activation functions could give contradictory reflex actions, for example, during eccentric contractions, where the human’s muscle is lengthened while tension is produced (e.g., slowly lowering a weight). Future investigation should be designed to determine when or how to choose one over the other.

Our future work will also focus on enhancing the resolution of the equilibrium-point controller. In order to accomplish this, new innovations are necessary to extend the bandwidth of the actuators, which would allow for a reduced refractory period in our algorithm and a smaller jump parameter Δ*P*, because with a shorter refractory period, the reflex frequency can be increased. We accomplished the equilibrium-point controller with only a few low-cost sensors and simple automata in the form of finite-state machines, minimizing the requirement for sophisticated electromechanical sensors. This lays the foundation for producing affordable, mass-producible wearable robots. We envision extending the equilibrium-point controller to multiple joints, including modules for the shoulder (Simpson et al., 2017; Zhou et al., 2021) and elbow (Koh et al., 2017) and adapting the technique for rehabilitation *via* preprogrammed trajectories (Chaparro-Rico et al., 2020).

Another direction is developing an analytical model *via* hybrid dynamical systems theory to understand the stability bounds in terms of the equilibrium-point controller parameters. A similar model has been applied to proprioceptive soft grippers (Park et al., 2021). Additionally, a more comprehensive study with human participants, including the participation of control subjects, would provide further opportunities for understanding the device’s influence on muscle coordination, skill acquisitions, and object manipulation.

## 5 Conclusion

In this study, we presented a soft wearable robot with a novel equilibrium-point controller intended to provide physical assistance to the forearm rotations of children with motor impairments. Through an experiment with human participants that consisted of trajectory tracking in different dynamic environments, the wearable robot demonstrated the ability to be volitionally controlled by all the users, which was achieved through triggered shifts in the robot’s equilibrium position. We analyzed the data with two linear mixed-effects models: one with participant effort as the outcome and the other with participant error as the outcome. The results provide a strong indication that the wearable robot reduces user effort. In addition, the models provided insight into the other main factors affecting effort and error, including the participant-specific capabilities and learning over time. Although the resulting physical assistance significantly reduced the required effort to complete the tracking task, it also significantly increased the error. We speculate that this is most likely due to the coarse nature of the assistance, which is a limitation of the prototype device. We envision several future advancements to the equilibrium-point control method, including the possibility of general impedance modulation capabilities. In summary, the equilibrium-point control method provides a new framework for facilitating human–robot interactions.

## Data Availability

The raw data supporting the conclusion of this article will be made available by the authors without undue reservation.

## Footnotes

1 Both authors were previously affiliated with the University of Southern California, where the studies took place.

2
https://github.com/jonreal/openWearable/tree/equilbrium-point-study.
