# Supplementary material for: Assisting Forearm Function in Children With Movement Disorders via A Soft Wearable Robot With Equilibrium-Point Control
Source: Front Robot AI. 2022 Jun 15;9:877041. doi: 10.3389/frobt.2022.877041 (PMC9240630; doi:10.3389/frobt.2022.877041)
Supplement: Supplementary file 1 [file DataSheet1.PDF]

Supplementary Material, Realmuto et al., Equilibrium-Point Soft Wearable Robot

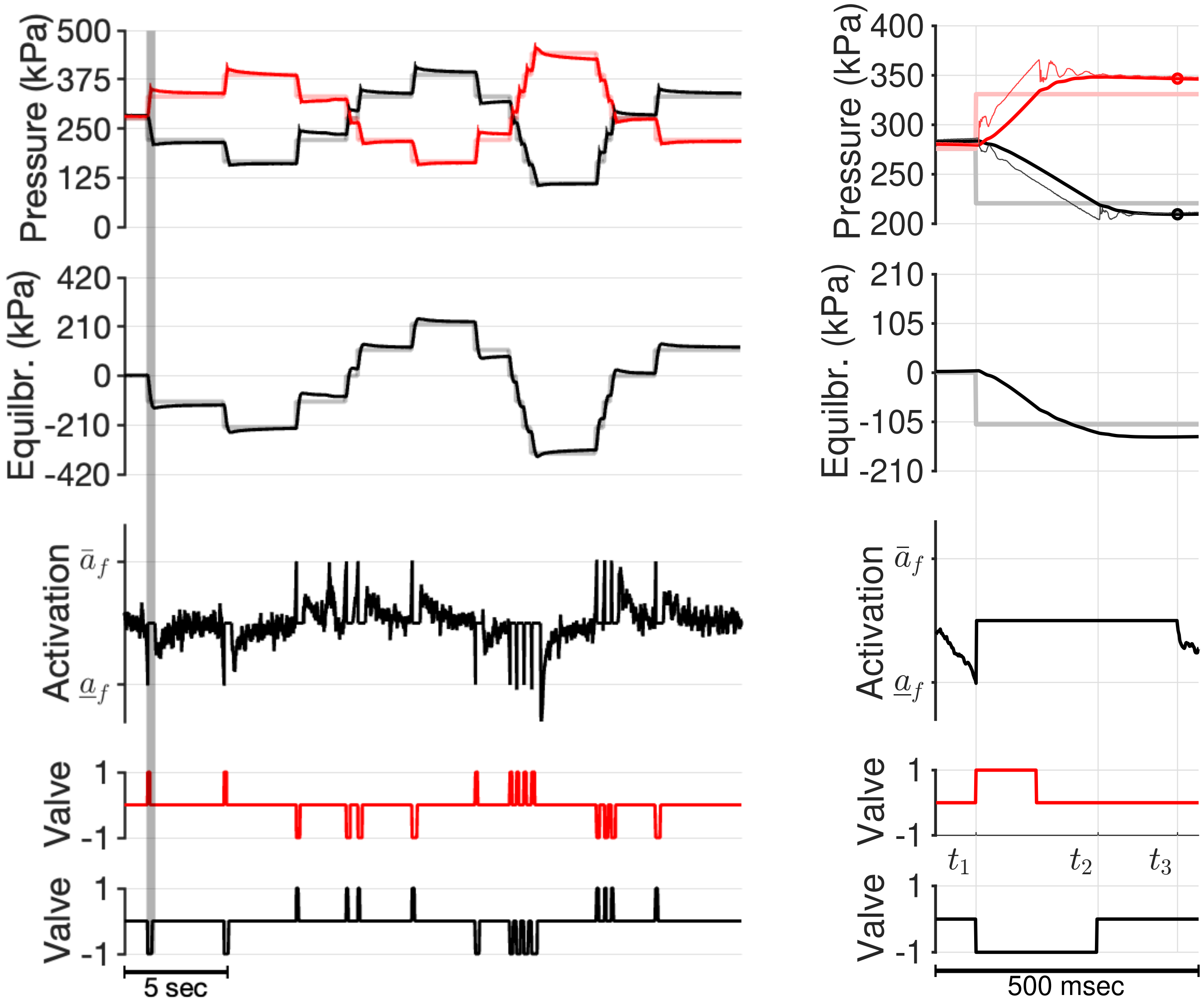

Figure S1. Left: Thirty seconds of signal traces during the interaction demo (Supplemental Material Video 1). Traces from top to bottom: actuator pressures, equilibrium position (calculated as the pressure difference between the antagonist actuators), activation signal, and both valve states (1 for 'inflate' and -1 for 'deflate'). A reflex (shift in equilibrium) is triggered when the activation signal crosses either threshold ($\bar{a}_f$ or $\underline{a}_f$). Right: Fifty millisecond zoom-in of the first reflex denoted as thin grey rectangle in left plots. Raw pressure signals can be seen as oscillatory responses, desired pressure signals ($P_{g,d}$ and $P_{n,d}$ in Eq. (4)) can be seen as step signals, and the estimated (low-pass filtered) pressures ($\hat{P}_g$ and $\hat{P}_n$ in Eq. (1)) are seen as the smoothed versions of the raw pressure signals. The threshold crossing (start of the reflex) is denoted by t_1. Time t_2 marks the start of the refractory period, which lasts until t_3, e.g., $T^* = t_3 - t_2 = 150$ msec (see Fig. 3 **B**). The new nominal pressures ($P_{g,0}$ and $P_{n,0}$ in Eq. 1) are denoted with circles. Note that the activation signal is $a = 0$ during the reflex.

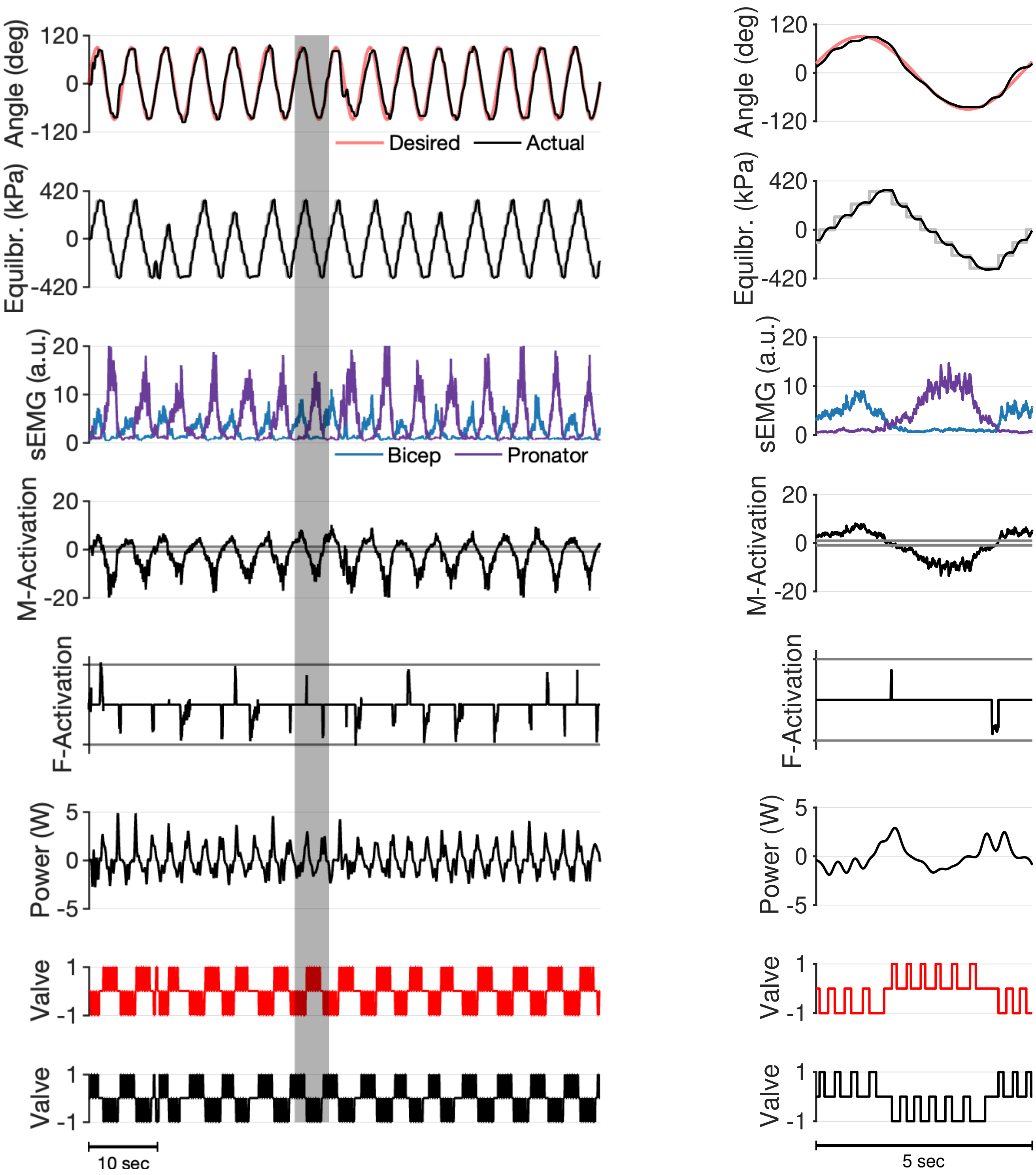

Figure S2. Left: Traces from an experimental trial (Participant P7, Block 1, Trial 9, *assist/Stiffness-Dominant*). Traces from top to bottom: desired and actual angular positions, equilibrium-point of the robot (calculated as the pressure difference between the antagonist actuators), M-Activation signal (Eq. (2)) with thresholds denoted as grey horizontal lines (± 1), F-Activation signal (Eq. (1)) with thresholds denoted as grey horizontal lines, interaction power (calculated as the product of the angular velocity of the haptic interface and the torque command of the haptic interface), and both valve states (1 for 'inflate' and -1 for 'deflate'). Right: Zoom-in of one cycle, which is denoted as grey rectangle in left plots.

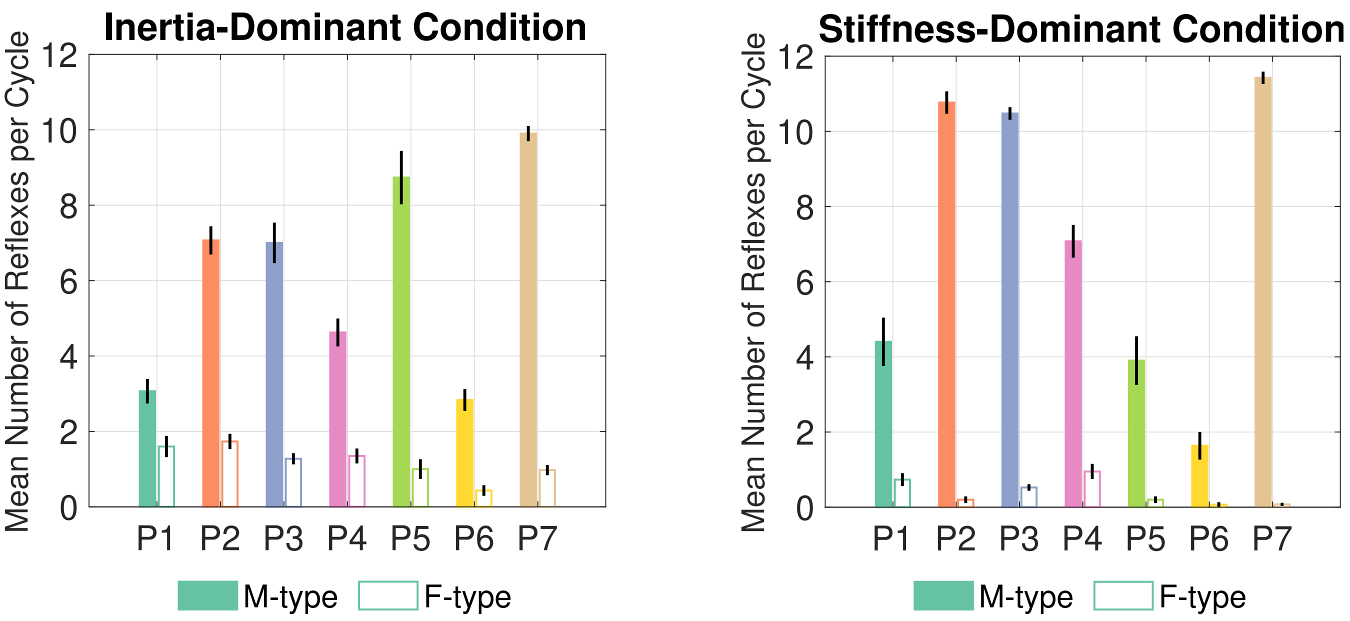

Figure S3. Mean number of reflexes during the *assistance* condition for each type of reflex during both haptic environments. Error bars correspond to standard error of the mean.

Table S1. Mean (std) error and effort outcome measured during the *Inertia-Dominate* environments. P-value corresponds to Kolmogorov–Smirnov test with the hypothesis that the undying distributions, for effort and error, differed between the *baseline* and *assistance* conditions

	Error			Effort		
Participant	Baseline	Assistance	p	Baseline	Assistance	p
P1	0.263 (0.110)	0.366 (0.273)	0.10884	0.347 (0.275)	0.129 (0.098)	0.0017459
P2	0.363 (0.250)	0.389 (0.204)	0.34203	0.376 (0.219)	0.197 (0.123)	0.00061578
P3	0.285 (0.210)	0.513 (0.200)	4.7066e-05	0.459 (0.206)	0.49 (0.281)	0.23074
P4	0.275 (0.215)	0.274 (0.152)	0.23074	0.568 (0.231)	0.279 (0.184)	1.5787e-05
P5	0.263 (0.213)	0.449 (0.209)	0.0017459	0.342 (0.170)	0.425 (0.236)	0.34203
P6	0.272 (0.157)	0.402 (0.270)	0.011314	0.579 (0.170)	0.401 (0.227)	0.00061578
P7	0.200 (0.137)	0.26 (0.193)	0.13925	0.507 (0.221)	0.21 (0.141)	4.3276e-07
Overall	0.215 (0.163)	0.278 (0.198)	0.00154	0.394 (0.235)	0.32 (0.209)	8.8583e-08

Table S2. Mean (std) error and effort outcome measured during the *Stiffness-Dominate* environments. P-value corresponds to Kolmogorov–Smirnov test with the hypothesis that the undying distributions, for effort and error, differed between the *baseline* and *assistance* conditions

	Error			Effort		
Participant	Baseline	Assistance	p	Baseline	Assistance	p
P1	0.228 (0.184)	0.267 (0.179)	0.10884	0.609 (0.210)	0.473 (0.250)	0.10884
P2	0.447 (0.218)	0.392 (0.236)	0.20033	0.499 (0.262)	0.501 (0.155)	0.20033
P3	0.232 (0.200)	0.234 (0.173)	0.72367	0.348 (0.240)	0.494 (0.260)	0.043135
P4	0.241 (0.192)	0.302 (0.213)	0.36131	0.387 (0.215)	0.334 (0.236)	0.23074
P5	0.386 (0.167)	0.402 (0.254)	0.5372	0.557 (0.244)	0.374 (0.191)	0.025856
P6	0.228 (0.130)	0.158 (0.185)	0.025856	0.358 (0.265)	0.211 (0.145)	0.20033
P7	0.193 (0.148)	0.26 (0.213)	0.13925	0.489 (0.196)	0.182 (0.098)	8.4173e-14
Overall	0.189 (0.141)	0.199 (0.154)	0.91851	0.294 (0.169)	0.248 (0.126)	7.1041e-07

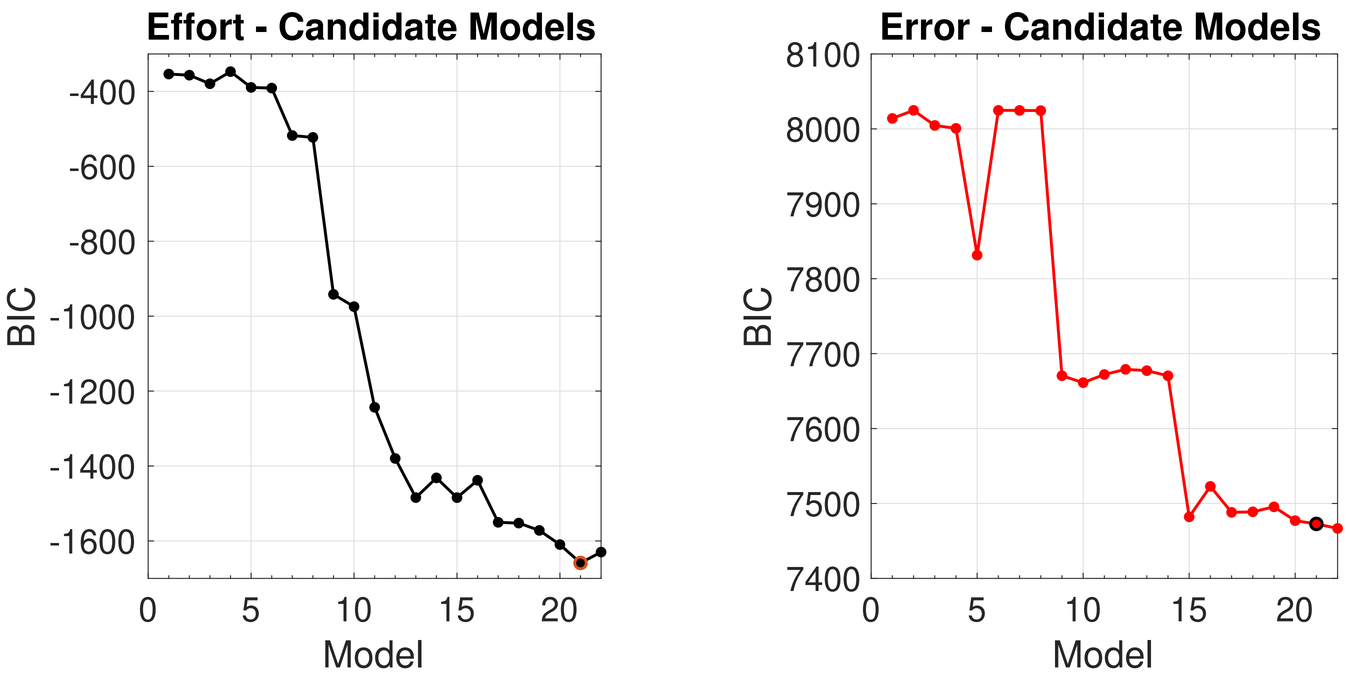

Figure S4. Bayesian information criterion for each candidate model. Model formulas are provided in Table S3.

Table S3. Candidate model formulas. Outcome variable is either error or effort. Corresponding Bayesian Information Criterion is shown in Fig. **??**

Model	Formula
1	y ∼ 1 + assist
2	y ∼ 1 + cycle number
3	y ∼ 1 + mreflex + freflex
4	y ∼ 1 + assist
5	y ∼ 1 + ID
6	y ∼ 1 + block
7	y ∼ 1 + power
8	y ∼ 1 + dynamic
9	y ∼ 1 + (1|ID)
10	y ∼ 1 + assist + (1|ID)
11	y ∼ 1 + power + (1|ID)
12	y ∼ 1 + power + (1|ID) + (1|dynamic)
13	y ∼ 1 + power + assist + (1|ID) + (1|dynamic) + (1|block)
14	y ∼ 1 + power + assist + (1|ID) + (1|dynamic)
15	y ∼ 1 + power + assist + (1|ID:dynamic) + (-1 + assist|ID:dynamic)
16	y ∼ 1 + power + mreflex + freflex + (1|ID:dynamic) + (-1 + mreflex|ID:dynamic) + (-1 + freflex|ID:dynamic)
17	y ∼ 1 + power + assist + block + (1|ID:dynamic) + (-1 + assist|ID:dynamic)
18	y ∼ 1 + power + assist + cycle number + (1|ID:dynamic) + (-1 + assist|ID:dynamic)
19	y ∼ 1 + power + assist + cycle number^2 + (1|ID:dynamic) + (-1 + assist|ID:dynamic)
20	y ∼ 1 + power + assist + cycle number + (1|ID:dynamic) + (-1 + assist|ID:dynamic) + (-1 + cycle number|ID)
21	y ∼ 1 + power + assist + cycle number + (1|ID:dynamic) + (-1 + assist|ID:dynamic) + (-1 + cycle number|ID:dynamic)
22	y ∼ 1 + power + assist + block + (1|ID:dynamic) + (-1 + assist|ID:dynamic) + (-1 + block|ID:dynamic)

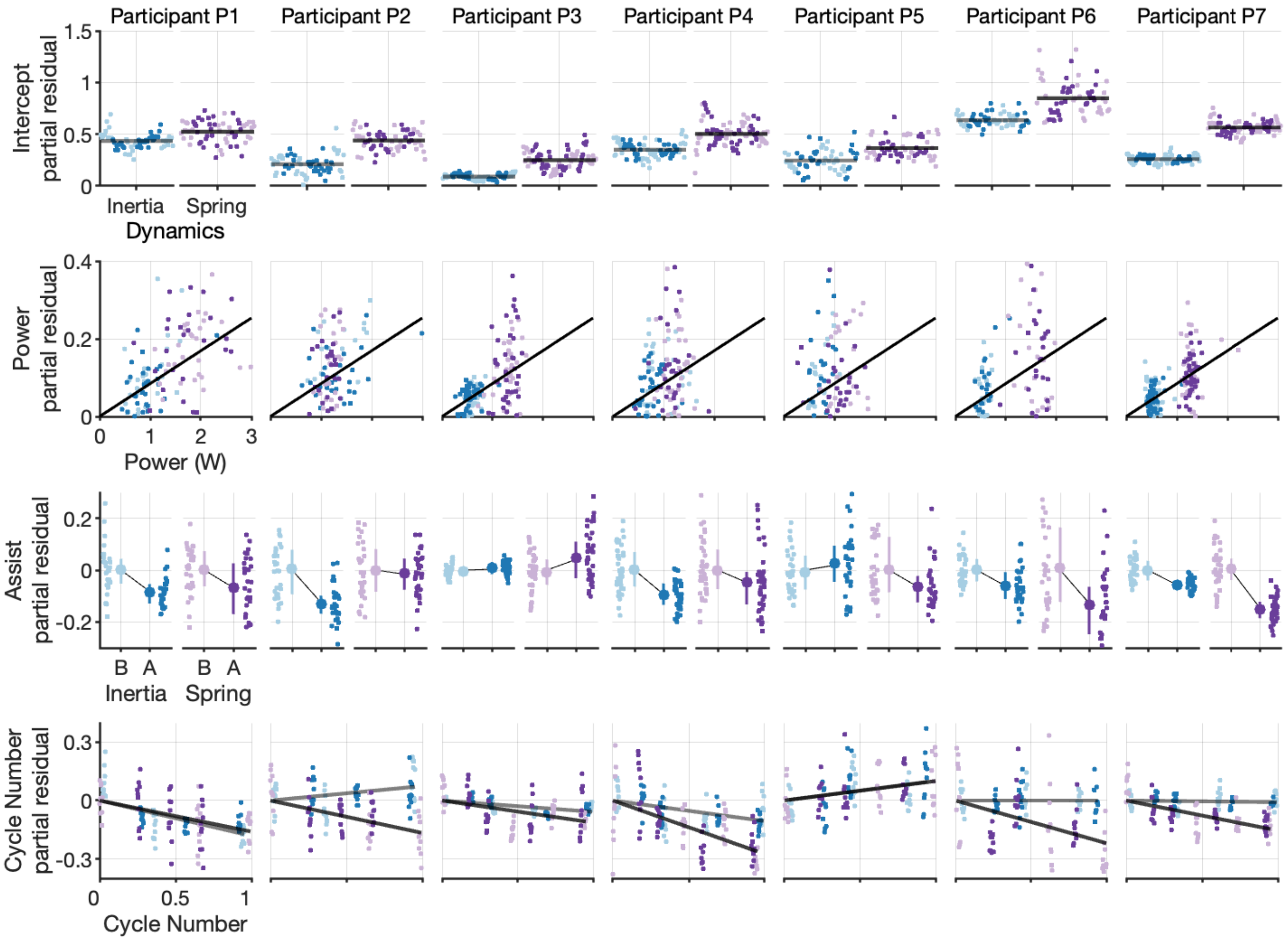

Figure S5. Effort partial residual (fixed and random effects) plot for each independent variable.

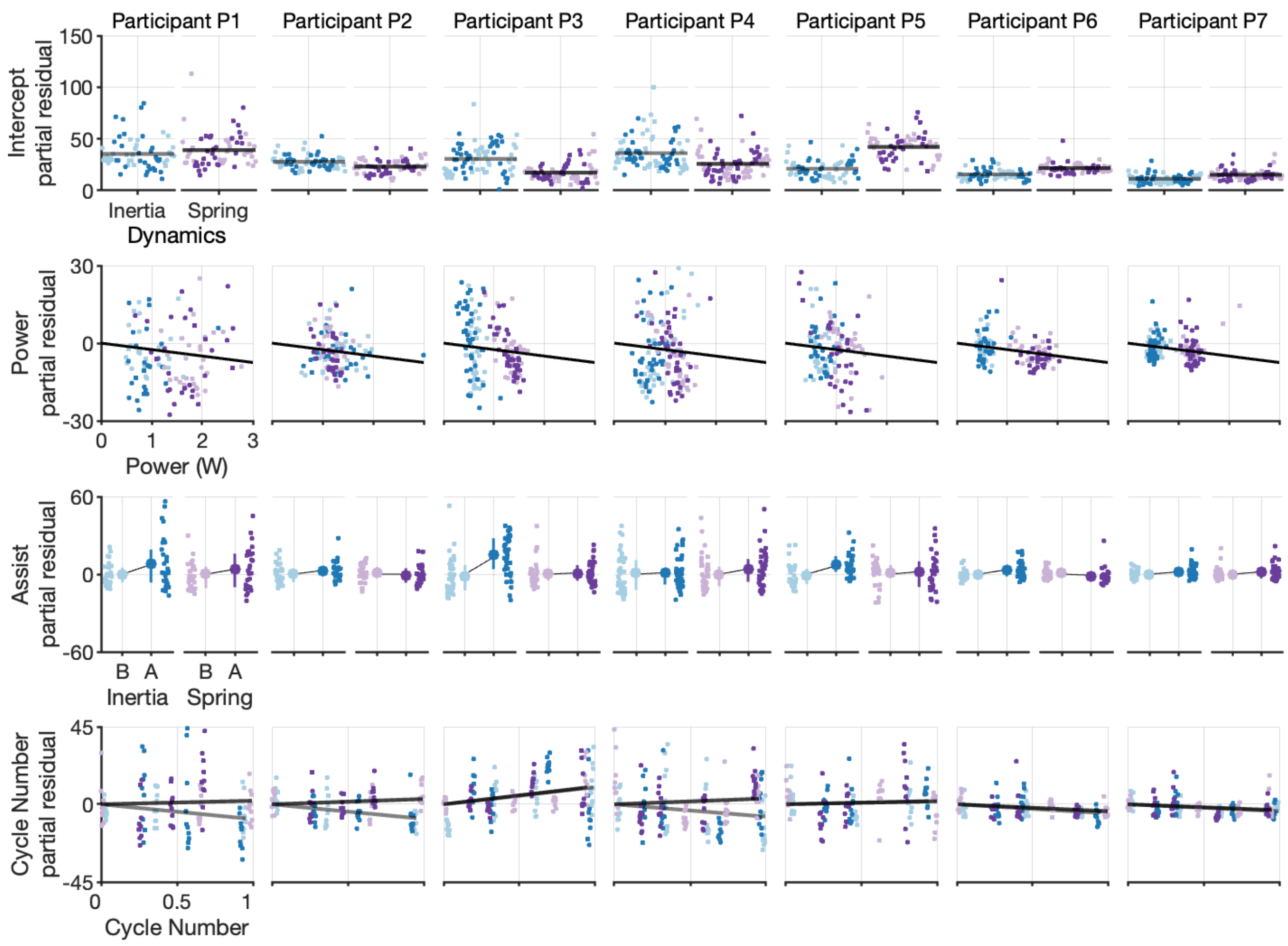

Figure S6. Error partial residual (fixed and random effects) plot for each independent variable.
